# Chromatin and Epigenetic Dysregulation of Prostate Cancer Development, Progression, and Therapeutic Response

**DOI:** 10.3390/cancers13133325

**Published:** 2021-07-02

**Authors:** Konsta Kukkonen, Sinja Taavitsainen, Laura Huhtala, Joonas Uusi-Makela, Kirsi J. Granberg, Matti Nykter, Alfonso Urbanucci

**Affiliations:** 1 Prostate Cancer Research Center, Faculty of Medicine and Health Technology, Tampere University and Tays Cancer Center, 33520 Tampere, Finland; konsta.kukkonen@tuni.fi (K.K.); sinja.taavitsainen@tuni.fi (S.T.); laura.huhtala@tuni.fi (L.H.); joonas.tuominen@tuni.fi (J.U.-M.); kirsi.granberg@tuni.fi (K.J.G.); matti.nykter@tuni.fi (M.N.); 2 Department of Tumor Biology, Institute for Cancer Research, Oslo University Hospital, 0424 Oslo, Norway

**Keywords:** prostate cancer, epigenetics, chromatin, lineage commitment, lineage plasticity, chromatin-associated factors, castration resistant prostate cancer, drug resistance, androgen receptor signaling inhibitors, chromatin regulators alterations

## Abstract

**Simple Summary:**

Chromatin and epigenetic alterations in cancer are responsible for a wide range of transcriptional changes that link DNA mutations to tumor phenotype. In this review, we explore studies describing recurrent epigenetic alterations in prostate cancer and highlight changes that occur during prostate carcinogenesis and progression to lethal treatment-resistant disease.

**Abstract:**

The dysregulation of chromatin and epigenetics has been defined as the overarching cancer hallmark. By disrupting transcriptional regulation in normal cells and mediating tumor progression by promoting cancer cell plasticity, this process has the ability to mediate all defined hallmarks of cancer. In this review, we collect and assess evidence on the contribution of chromatin and epigenetic dysregulation in prostate cancer. We highlight important mechanisms leading to prostate carcinogenesis, the emergence of castration-resistance upon treatment with androgen deprivation therapy, and resistance to antiandrogens. We examine in particular the contribution of chromatin structure and epigenetics to cell lineage commitment, which is dysregulated during tumorigenesis, and cell plasticity, which is altered during tumor progression.

## 1. Introduction

**Figure 1:**
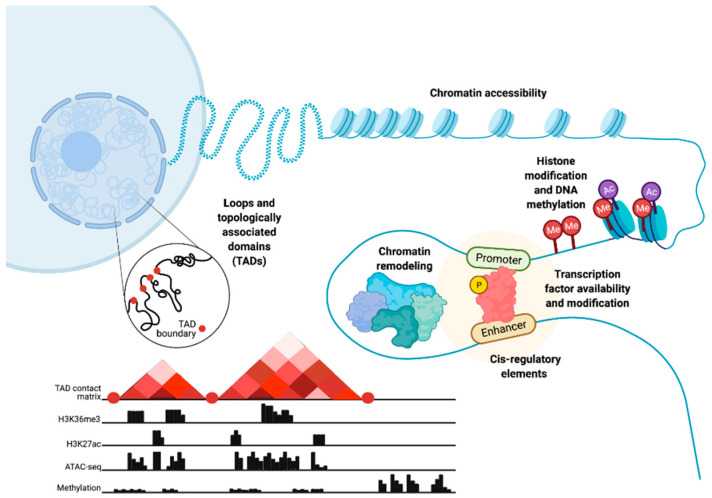
Epigenetic- and chromatin-related mechanisms with potential for dysregulation in prostate cancer cells. Epigenetic dysregulation can occur at multiple levels, including changes in chromatin accessibility, histone and DNA modification through processes such as methylation, chromatin remodeling, modification of transcription factors and changes in their availability, cis-regulatory elements, chromatin loops, and topologically associated domains. These chromatin and epigenetic features can be analyzed via the integration of multiple high-throughput sequencing data types. These data include chromatin conformation capture (Hi-C) to understand the 3D chromatin structure and topologically associated domains, chromatin immunoprecipitation (ChIP-seq) to study histone markers, assay for transposase-accessible chromatin (ATAC-seq) to show chromatin accessibility patterns, and DNA methylation sequencing. Figure created with BioRender.com.

Chromatin structure and epigenetics are intertwined but, nonetheless, distinct entities that have been implicated in prostate cancer (PC) disease initiation and progression. Here, we consider epigenetics to be the collection of DNA modifications such as DNA methylation. This definition of epigenetics can also include histone modifications and the binding of transcription factors (TFs) to DNA. Histone modifications are often referred to as epigenetic profiles as they determine chromatin states and nucleosome positioning, which, in turn, allows for DNA accessibility (Figure 1). Therefore, we additionally define chromatin structure as histone modifications and nucleosome positioning, as well as the three-dimensional (3D) organization of the chromatin within the nucleus (Figure 1).

PC continues to be a major cause of cancer-related death in men worldwide [1]. Although primary intervention with radiotherapy or surgery and androgen deprivation therapy (ADT) have a curative intent in hormone-sensitive PC (HSPC), metastatic disease remains incurable, despite the introduction of combination approaches [2]. Targeted systemic therapies with androgen receptor (AR) signaling inhibitors (ARSIs) such as abiraterone or enzalutamide are primarily used to treat relapsed castration-resistant prostate cancer (CRPC) [3]. Employment of these agents has been shown to be effective, especially in high risk primary metastatic HSPCs patients, and their use is becoming more common [4,5]. Although these combination approaches have demonstrated survival benefits, they have also been shown to contribute to the emergence of more aggressive castration-resistant tumors [6]. The majority of castration- and some ARSI-resistant PCs are characterized by increased AR signaling [7,8]. However, alternative forms of castration-resistance have also been identified, including forms that are AR-negative with neuroendocrine-like features [9], forms indifferent to AR [10], and forms that are more dependent on alternative signaling pathways such as fibroblast growth factor (FGF) [11]. Additionally, forms of castration-resistance dependent on other TFs such as the glucocorticoid receptor (GR) or the pluripotent stem cell TF SOX2 [12,13,14,15] have been described.

Along with others, we previously reported that the emergence of castration-resistance and AR overexpression are associated with chromatin reprogramming [16,17,18,19]. As with other malignancies, PC is thought to arise from and be driven by oncogenic genetic alterations. However, as with many cancers, PC cannot be explained solely on the basis of genetic alterations [20]. PC in particular has a relatively low mutational load at presentation [21] and prostate carcinogenesis is not clearly driven by any particular genetic alterations [22]. Nevertheless, HSPCs and CRPCs are characterized by the typical cancer hallmarks [23,24] that mediate carcinogenesis, disease progression under treatment pressure, and cancer growth beyond the tumor microenvironment (TME).

Flavahan and colleagues first proposed the concept of epigenetic plasticity, by which alterations in the structure of the chromatin, or chromatin states, and epigenetic alterations would be able to confer the full range of cancer hallmarks by altering transcriptional regulation [25,26]. Indeed, many of the epigenetic and chromatin regulators that drive normal tissue development and differentiation are well-known oncogenes and tumor suppressors recurrently mutated or aberrantly expressed in different malignancies.

Similarly, it is well known that cellular lineage identity is defined by tightly regulated chromatin-related processes and the epigenetic landscape [27]. The concept of lineage plasticity is of clinical relevance for PC as it is a common mechanism of resistance following the increasing usage of more potent ARSI in primary disease [11,28].

Due to the dynamic sets of chromatin and epigenetic alterations and their reversibility, such alterations can play a significant role in driving both carcinogenesis and progression to treatment-resistant disease. These alterations can also be stochastically different in individual cells [25]. Therefore, epigenetic plasticity can be the basis of the heterogeneity observed among PC patients, but also between different tumor foci of the same prostate [29,30,31]. It is apt that epigenetic plasticity can consist of genetic changes that allow normal cells to transform through alteration of lineage commitment and further allow malignant cells to adapt under diverse treatment pressure by mediating lineage plasticity.

**Figure 2:**
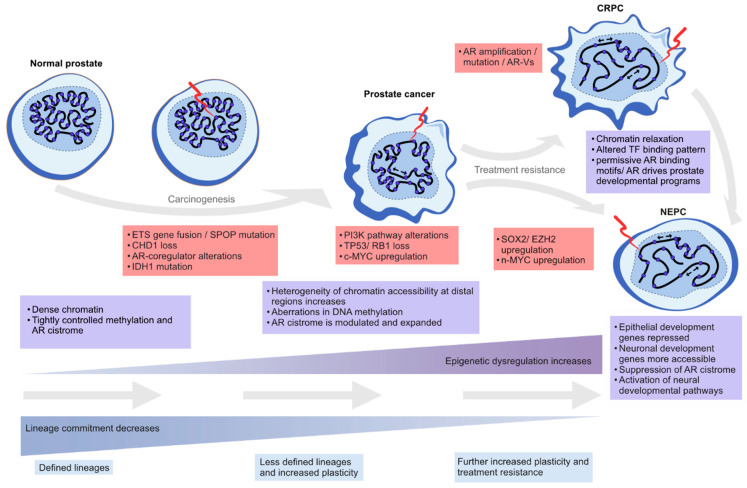
Epigenetic plasticity in prostate cancer. Epigenetic dysregulation (light blue boxes) is in the forefront of lineage plasticity as well as in carcinogenesis and therapy resistance. Normal prostate epithelium is renewing at a steady state as terminally differentiated luminal cells are slowly replaced by progenitor cells. As genetic alterations accumulate due to cell division and the normal aging process, driver alterations (red boxes) such as *ETS* gene fusions or *SPOP* mutations emerge. The mutational processes lead to less ordered chromatin structure, as characterized by chromatin relaxation at distal regulatory regions, alterations in DNA methylation and histone modifications, and dysregulation of higher order chromatin structures, which alters the binding of key TFs such as AR. Some cells gain stem-like properties, leading to increased proliferation capacity and reduced apoptotic rates, which leads to tumor formation over time. The plasticity of the cellular identity is also in a key role during the emergence of treatment resistance as the cancer cells can repurpose differentiation-promoting transcription factors such as AR into regulatory regions supporting developmental gene expression (seen in castration-resistant prostate adenocarcinoma, CRPC), or transdifferentiate into non-luminal, small cell prostate carcinoma or neuroendocrine prostate cancer (NEPC).

In this review, we highlight the contribution of chromatin- and epigenetics-related processes to prostate carcinogenesis and progression to treatment resistance. We explore the role of epigenetic regulation and TFs in lineage commitment and plasticity in the normal prostate and PC cells, the mutations and the altered expression of key genes coding for chromatin-associated proteins, alterations in DNA methylation patterns, and changes in the structure and 3D organization of the chromatin (Figure 2).

## 2. Prostate Lineage Commitment and Prostate Cancer Plasticity

Here, we collect evidence of the involvement of lineage commitment and cancer cell plasticity in PC initiation and resistance to treatment, respectively.

The normal epithelium of the prostate secretory acini is composed of a layer of luminal cells oriented towards the acinar lumen and a basal cell layer that also includes rare neuroendocrine cells. The determinants of prostatic epithelial differentiation include master regulators such as AR, FOXA1, and NKX3-1. Talos and colleagues showed that these TFs were sufficient and required for differentiation of iPS cells of mouse fibroblast origin in prostatic cells engrafted in the renal capsule [32]. Xie et al. showed that AR may also be required in the basal-luminal intermediate cells to produce fully differentiated luminal progeny in adult mice [33]. The same study found that AR expression is not needed for survival of the luminal cells but is essential for normal luminal differentiation and morphology [33]. This may be, in part, due to an indirect effect by *NKX3-1*, as it is an AR target gene and was not expressed after deletion of AR [33]. Dutta et al. had previously shown that NKX3-1 is a prostate-specific master transcriptional regulator that can transdifferentiate the seminal vesicle epithelium into prostate epithelium [34]. Of note, the seminal vesicle epithelium expresses AR and FOXA1 following the introduction of NKX3-1 [34]. Differentiation by NKX3-1 was found to be mediated by histone demethylase UTY and histone methylase G9a [34]. The stromal component seems to contribute to the re-differentiation process as the urogenital sinus mesenchyme is always co-transplanted with the epithelial cells in these experiments [32,34].

AR and FOXA1 have been found to also be important for the regulation of the Homeobox (HOX) A genes [35]. The HOX genes regulate prostate development in mice [36] and the paralogous HOX13 genes (*HOXA13*, *HOXB13*, and *HOXD13*) are still expressed in the luminal epithelium of the human adult prostate [36]. In recent single cell analyses, Guo et al. showed that terminally differentiated luminal cells express *NKX3-1* and *HOXB13* together with *AR* [37].

Experiments in mice have shown that the prostate epithelium displays a regenerative capacity following repeated cycles of androgen deprivation [38,39]. This led to the hypothesis that the prostate epithelium may also harbor stem cells responsible for tissue renewal [38]. Recent advances in single cell sequencing have facilitated a more detailed characterization of the complexity of prostate cell types without having to rely on restricted sets of cell surface markers for their classification [40]. These single cell approaches have led to the identification of club cells (KRT5^−^, KRT8^−^, and SCGB1A1^+^) and hillock cells (KRT5^+^, KRT14^−^, KRT13^+^) with stem cell potential [40]. Similar to lung club and hillock cells, progenitor-like cells that are able to differentiate into goblet cells and ciliated cells, these newly described prostate cells are efficient at reconstituting the prostate epithelium in in vivo studies following androgen deprivation, as shown by Karthaus et al. [41], although their transdifferentiation to basal cells is rather limited [40,41,42].

Maitland and Collins have reviewed how the prostate epithelium constantly renews and the terminal differentiation of luminal cells is associated with a higher rate of apoptosis [43]. The existence of intermediate cells, which are cells with both basal and luminal features in the prostate epithelium, has been acknowledged for a long time [44]. In lineage tracing studies with mice, both basal and luminal cells have been shown to contribute to the renewal of prostate epithelium during androgen deprivation and add back cycles, indicating that there are several degrees of stemness within the adult prostate epithelium [37,41,45]. In previous studies, both luminal and basal stem or progenitor cells have been shown to act as the PC-initiating cells, but PC is generally characterized by the absence of basal cells [45,46,47,48,49,50].

Lineage plasticity refers to the reversal of the process of lineage commitment, either by dedifferentiation of the more differentiated cells or, in extreme cases, transdifferentiation directly (or via an intermediate) to another epithelial cell lineage. This is a process that is unlikely to occur in the normal prostate under physiological conditions. However, pathological processes or stressors such as inflammation may alter this scheme [51]. Alterations in key epigenetic- and chromatin-associated or environmental stress factors can disrupt the epigenetic homeostasis of normal cells, which may lead to lineage plasticity, differentiation arrest, and accumulation of undifferentiated cells in transition. The role of the TME in determining such alterations is not well known, but factors such as hypoxia [52] and other metabolic stressors [53] have been associated with more aggressive PC phenotypes. A well-established example of metabolic stress leading to epigenetic changes is the dysregulation of one-carbon metabolism and its effects on both DNA and histone methylation [53,54].

Lineage plasticity during prostate carcinogenesis is poorly understood. However, for example, the upregulation of c-MYC is a common early event [55] and has been implicated in the gain of stem cell properties and repression of differentiation [56]. Normal luminal cells repress MYC expression via AR/β-catenin/TCF-4 signaling in the presence of androgens, leading to growth arrest, but overexpressing c-MYC rescues cell proliferation [57,58]. This highlights the opposing roles of AR as the main differentiation factor and growth suppressor in the non-transformed secretory luminal prostate cells and as a prominent driver of PC cells’ proliferation, as discussed below.

Cells with regenerative potential within the normal prostate tissue have been hypothesized to be the cells of origin for PC [59]. Indeed, Song et al. recently described club-like PC cells in primary PC specimens [60]. These cells are transcriptionally similar but have higher AR expression and an enhanced androgen signaling signature when compared to club cells from normal prostates [60] (Table S1). The overexpression of AR in these cells is consistent with the modulation and expansion of the AR cistrome, which is a well-documented feature of PC initiation [61]. Under this scenario, AR overexpression induces changes in the AR transcriptional program, leading to cell survival and proliferation, possibly via the alteration of the pioneer activity of cooperative TFs of the AR such as HOXB13 [61]. Interestingly, recent analyses with mice expressing the F133V mutant form of Speckle Type BTB/POZ Protein (*SPOP*) showed that this mutation is sufficient to modify chromatin accessibility and binding of AR and FOXA1 at PC specific genomic sites [61,62]. Further expansion of the AR cistrome has also been reported during progression to CRPC [63,64].

The role of some luminal progenitor cells as possible cells of origin for PC is also supported by studies in Tmprss2-CreER;Pten^flox/flox^ mice [37]. The TACSTD2/Trop2 (encoded by *Tacstd2*)-expressing luminal progenitor cells characterized by Guo et al. have similar transcriptional features to the club cells characterized by Henry et al., Karthaus et al., and Song et al., based on the presence of markers such as *PIGR*, *PSCA*, and *KRT4* [37,40,41,60] and high expression of TACSTD2 [41,60] (Table S1). Similarly, Kwon et al. found that TACSTD2+ luminal prostate cells were more efficiently transformed in vitro in an organoid-forming assay than TACSTD2−luminal cells [65]. The authors also showed that TACSTD2+ luminal cells express SOX2 and display remarkable plasticity by transdifferentiation to de novo neuroendocrine PC (NEPC), even in the absence of selection pressure from treatment with antiandrogens [65].

Although the above-described studies suggest that multiple subpopulations of cells can give rise to PC [37,45,46,47,48,50], their transcriptional features seem to converge toward features of luminal progenitor or club-like cells [37,41,48,49,60]. Undoubtedly, the cell type of origin of PC can have clinically relevant consequences in terms of tumor trajectory and prognosis [50].

Treatment of metastatic HSPCs with ARSI in combination with ADT imposes a strong negative selection pressure on the cancer cell population. Multiple treatment resistance mechanisms arise from genetic and epigenetic alterations, leading to a rewiring of alternative bypass pathways driving tumor cell growth [66], but the majority of CRPCs remain AR-dependent through maintenance of AR signaling [67]. This imposes chromatin reconfiguration and epigenetic plasticity in ARSI-resistant PC cells, leading to heterogeneous CRPC phenotypes more or less dependent on AR signaling, with both luminal and basal features [68,69,70]. A recent single cell analysis by He and colleagues showed that different AR transcript variants are ubiquitously expressed prior to treatment with ARSI [71]. They also found that ARSI drives resistance pathways such as epithelial to mesenchymal transition (EMT), a form of lineage plasticity generally associated with metastatic disease [71].

Chromatin reprogramming is a key feature of lineage plasticity during treatment resistance, for example by allowing reactivation of pathways that are normally active only during prostate tissue development [19,64,72]. Zhang et al. found that loss of the chromatin-modifying helicase CHD1 confers enzalutamide resistance by inducing marked changes in chromatin accessibility, along with transcriptional rewiring and upregulation of TFs *NR3C1* (GR), *POU3F2* (BRN2), *TBX2*, and *NR2F1*, leading to gene expression changes including a reduction in luminal and an increase in EMT markers [68].

Cellular plasticity has also been associated with alterations in tumor suppressor genes and chromatin reconfiguration, exemplified by the deletion of *TP53* and *RB1* that can lead to treatment resistance by allowing for diverse transcriptional programs [6,69,70]. P53 and pRb (encoded by *TP53* and *RB1*, respectively) cooperate to suppress expression of *SOX2*, a well-known factor of pluripotency, which, in part, explains the association of these factors with lineage plasticity [15,73]. Using an isogenic model of HSPC (LNCaP) and CRPC (C4-2), Mandingo et al. showed that *RB1* loss leads to a reconfiguration of E2F activity for increasing the production of antioxidants, protecting cells against doxorubicin in the CRPC cells that is not observed in the HSPC cells [74]. These findings highlight how transcriptional reprogramming by the same TF can be modulated in different disease stages [74].

In the context of ARSI resistance, so-called treatment-induced neuroendocrine prostate cancers (t-NEPCs) (reviewed by Rubin et al. and Kaarijärvi et al. [28,75]) represent a tumor phenotype mediated by chromatin and transcriptional plasticity, which, in turn, drives cellular plasticity. During therapies with ARSIs, AR-dependent cells are depleted, while transcriptionally and epigenetically heterogeneous AR-indifferent cells increase their growth and survival potential [11,68,69]. Increased mutation frequency in tumor suppressor genes such as *TP53* and *RB1* suggests that t-NEPCs require these alterations for tumor selective reprogramming [6,15,73,76]. The t-NEPC tumors acquire neuroendocrine features and suppress luminal transcriptional programs via the upregulation of SOX2 and EZH2 in mouse models [15,73] and the upregulation of *LHX2* and *ISL1* in human cells, which goes hand in hand with increased chromatin accessibility and the transcriptional output of neuroendocrine lineage-specific genes [76]. FOXA1, a well-known AR pioneering factor and a mediator of the transcriptional output in prostate adenocarcinoma, has also been shown to mediate NE-specific transcription in NEPC [77]. NE-specific genes are repressed in normal differentiated prostate epithelial cells, but during transdifferentiation following the activation of NE TFs such as ASCL1 and NKX2-1 in these cells, the chromatin structure in their proximal regulatory regions is rewired and allows for their expression [77]. The loss of the transcriptional repressor REST, which normally represses *ASCL1* and *NKX2-1*, has been implicated in this process [78]. Active AR signaling keeps REST stable via inhibition of E-ubiquitin ligase β-TrcP, so ARSIs may contribute to the downregulation of REST [79].

Ultimately, PC progression is a continuous process resulting from the selection pressures of treatment upon the tumor, the TME, and the host immune system [80].

## 3. Mutations and Expression Dysregulation of Genes Coding for Chromatin-Associated Factors

Many studies have illustrated how chromatin-associated factors affect both lineage commitment and lineage plasticity, which is likely to drive tumor progression [64,68,77]. PC cell lineage plasticity is driven by genetic alterations, gene expression changes, and the altered activity of chromatin-associated and epigenetic regulators [6,25,81,82,83,84]. These chromatin-associated factors can be broadly categorized by their protein function into TFs, transcriptional co-regulators, chromatin modifiers, and genes involved in mRNA transcript synthesis or processing. In this section, we performed an analysis of their mutational and expression patterns in prostate carcinogenesis and the development of treatment resistance. To gain a comprehensive view on chromatin-associated factors, we utilized a list of 2754 genes previously annotated to the above-mentioned functional groupings [85] and queried their alteration status in publicly available PC patient datasets.

Somatic mutation data from the International Cancer Genome Consortium (ICGC) prostate adenocarcinoma datasets [86] showed that only 34 were genes mutated in more than 1% of patients with potential protein function-altering effects, reflecting the overall low mutation frequency of these tumors (Table S2). As the ICGC cohort largely consists of early stage primary prostate tumors, we repeated the analysis using two metastatic CRPC patient cohorts from Robinson et al. and Grasso et al. to include 150 pre- or post-ARSI mCRPC biopsies [87] and 50 heavily pre-treated lethal CRPCs exposed only to first generation ADT [88], respectively. We identified an additional 78 genes coding for chromatin-associated proteins in more than 2% of these patients with protein-altering mutations (Table S2). Altogether, 18 genes, including *TP53*, *FOXA1*, *SPOP*, and *CDK12*, were recurrently mutated in both the ICGC early stage and advanced CRPC tumors. The proportions of mutated genes from different functional categories did not differ significantly in the early versus treatment-resistant disease stages.

**Figure 3:**
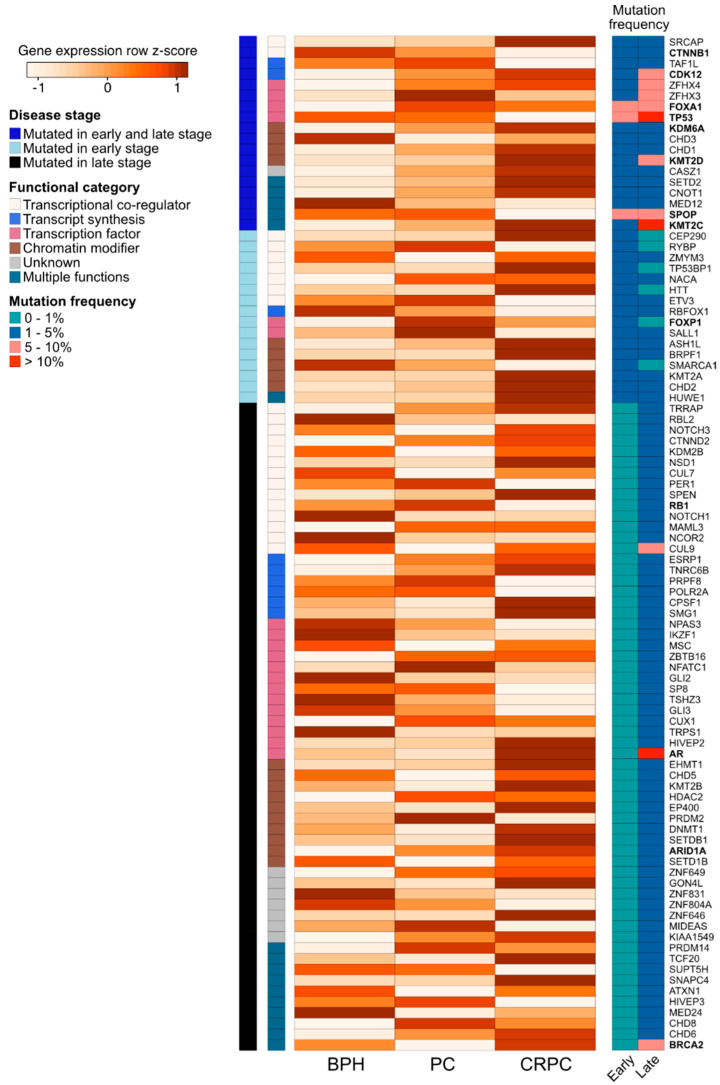
Expression changes of 94 recurrently mutated genes coding for chromatin-associated proteins in prostate carcinogenesis and development of treatment resistance. Row-scaled log2 mean expression values for each gene (the rows) are shown in a heatmap for benign prostatic hyperplasia (BPH), untreated prostate cancer (PC), and castration-resistant prostate cancer (CRPC) patient samples. The names of frequently studied genes are shown in bold. Each row is annotated with the disease stage, in which the gene is found to be recurrently mutated (either early stage (PC), late stage (CRPC), or both early and late stage). The rows are also annotated with the functional category of each gene. On the right, two columns show the mutation frequency of the gene in early- and late-stage disease in four categories (0–1%, 1–5%, 5–10%, and >10%).

We further assessed the expression of the 94 recurrently mutated genes (Table S2) during prostate carcinogenesis and the development of treatment resistance in a previously published RNA-sequencing dataset of benign prostatic hyperplasia (BPH, *n* = 10), untreated PC (*n* = 16), and CRPC (*n* = 11) samples (Figure 3) [89]. We observed expression changes in several known PC driver genes including the loss of expression of *TP53* and *SPOP* during the transition from untreated PC to CRPC, as well as the increased expression of *AR*, *BRCA2*, *KDM6A*, and *CDK12* in CRPC. Interestingly, TFs that were mutated in PC and CRPC or only in CRPC showed higher expression in those categories, with the exception of decreased *TP53* expression in CRPC, which is potentially a consequence of its wide-ranging tumor suppressive functions [70]. TFs mutated only in CRPC typically had lower expression in CRPC, with the exception of *AR* and *EHMT1*, a histone methyltransferase with potential tumor suppressive function in the prostate [90]. Nearly all recurrently mutated chromatin modifiers had the highest expression in CRPC irrespective of the mutation frequency (Figure 3). Among the genes involved in epigenetic, chromatin, or gene regulation that were not recurrently mutated, 68 (2.6%) genes were significantly upregulated during prostate carcinogenesis (PC vs. BPH), while 80 (3.0%) genes were upregulated during the development of treatment resistance (CRPC vs. PC). In contrast, 34 (1.3%) non-recurrently mutated genes were downregulated during prostate carcinogenesis and 77 (2.9%) were downregulated during treatment resistance. TFs were the most common group of genes to be aberrantly expressed, particularly amongst the genes upregulated during carcinogenesis (46 of 68 genes, 68%) or downregulated during carcinogenesis (18 of 34 genes, 53%).

Our analysis further highlighted alterations in groups of genes with functions related to PC lineage plasticity. These included the master transcriptional regulators *FOXA1* and *AR* [91,92,93,94], the *KMT2A-D* transcriptional co-activators involved in development [95], and a number of chromodomain (CHD) genes, including *CHD1*, involved in chromatin remodeling and transcription activation [96]. Loss of *CHD1* has been reported in 15% of HSPCs and 17% of CRPCs [97] and *CHD1* loss has been implicated in PC cell chromatin rewiring with tumor-suppressing functions [68] and increased sensitivity of PC tumors to DNA damage [98]. The simultaneous dysregulation of tumor suppressors such as *TP53* and *RB1* in our recurrently mutated gene list has also been shown to promote PC plasticity [99].

In addition to genetic variation in coding regions as explored here, recent studies have also shown that alterations within noncoding regions affect chromatin conformation and transcriptional regulation in PC. By studying somatic single nucleotide variants and germline single nucleotide polymorphisms in the *cis*-regulatory elements of prostate tumors, Mazrooei et al. found that these variants are specifically enriched in the cistromes of master transcriptional regulators AR, FOXA1, HOXB13, and SOX9 [100]. This implicates noncoding variation in the dysregulation of the chromatin binding activity of these factors and therefore, in prostate carcinogenesis. *AR* expression has further been found to be modulated through a somatically gained upstream enhancer in CRPC [101] and its binding sites have been shown to be lost or gained due to the activity of FOXA1 and HOXB13 during carcinogenesis [61]. The chromatin regions linked to altered gene regulation in PC have also been shown to be enriched in PC predisposing genetic variants. Pomerantz et al. analyzed prostate lineage-specific enhancers and promoters marked by a combination of histone modifications and PC-specific TFs AR, FOXA1, and HOXB13, and showed that these were enriched for genetic variants linked to increased PC risk, depicting an active epigenetic state [64]. In addition, they showed that prostate lineage-specific regulatory regions exhibit active epigenetic states that are predicted to increase somatic mutational burden [64]. Collectively, these studies highlight the importance of the noncoding genome in PC lineage commitment and plasticity.

## 4. The Role of DNA Methylation in Prostate Cancer

The role of DNA methylation in PC has been studied for several decades. A number of studies have found differences in DNA methylation between normal and tumor tissue, suggesting that these changes either contribute to prostate carcinogenesis or that such changes develop during carcinogenesis and are associated with lineage plasticity [102,103,104,105,106,107,108,109,110,111] via regulation of gene expression [81,112,113,114]. The effects of DNA methylation on gene expression observed in cancer can be ascertained to changes leading to silencing of the proximal genes but also to the possible impact that the modification of methylation would have on the ability of the DNA to bind histones, TFs, and chromatin-associated protein complexes, thereby inducing chromatin conformation changes and altering TF binding specificity [115].

A comprehensive meta-analysis of DNA methylation in PC has been reported by Massie et al. [116]. The meta-analysis further validated typically methylated genes such as *GSTP1* and *RARβ*, and provided some mechanistic rationale and a link to metabolic changes, possibly causing changes in methylation patterns during prostate carcinogenesis [116].

One of the first findings linking alteration of DNA methylation patterns to PC was reported by Lee et al. [102]. They showed that *GSTP1* was not expressed in PC tissue due to the hypermethylation of the promoter [102]. The *GSTP1* promoter is not methylated in BPH and the gene is also expressed in normal basal epithelial cells [102]. As *GSTP1* expression is already lost in prostatic intraepithelial neoplasia (PIN), luminal cells, and in glands from PC tissue, its use as an early carcinogenesis tissue marker has been suggested [103,104]. Subsequently, whole-genome methylation studies revealed changes in DNA methylation patterns during prostate carcinogenesis [105,106,107,108,109,110,111]. These studies confirmed the hypermethylation of *GSTP1* and revealed several other promoters of genes, including *RARβ, HIF3A* and *HAAO* with functions related to tumor suppression, response to hypoxia, and microsatellite stability, respectively, which were hypermethylated in PC when compared to benign tissue [105,109,110,117,118]. Similarly, studies in PC tissue showed hypermethylation of *HOXD3*, a gene involved in TGFβ signaling, and *BMP7,* which has been reported to suppress metastatic potential [110,119].

The combination of DNA methylation and gene expression changes in respective genes can also be used to predict treatment outcomes. Panja et al. showed that a panel of as little as five methylation sites and mRNA expression changes in their site-harboring genes could be used to predict response to ADT in the TCGA PC cohort [120].

Alterations in DNA methylation patterns in larger regions than promoters have also been linked to carcinogenesis. Fiano et al. showed that the hypomethylation of long interspersed nuclear element-1 (LINE-1), which represents about 15% of the human genome, is associated with PC mortality [121]. It was also previously shown that there is decreased DNA methylation in LINE-1 repetitive elements in ETS-negative cases, suggesting that this effect might be related to ETS status [109].

Although methylation of 5′ carbon of cytosine (5mC) is the most commonly studied form of DNA methylation, there are also other types of DNA modifications associated with gene expression regulation that are less studied in PC: 5-hydroxymethylcytosine (5hmC), 5-formylcytosine (5fC), and 5-carboxylcytosine (5caC), all formed from the 5mC oxidation process [122]. Storebjerg et al. showed that 5hmC levels are reduced in PC when compared to non-malignant prostate tissue, especially in ERG-negative tumors [122]. They also reported that 5fC levels are increased in ERG-positive tumors and that 5caC levels are elevated in all the PC samples examined in their study [122]. In addition, they were also able to connect 5hmC and 5caC levels to prognosis [122]. However, since this study performed immunohistochemistry (IHC) analysis, the genomic areas and the genes implicated by these less studied forms of DNA modification remain to be examined.

DNA methylation patterns have also been associated with specific genomic alterations in PC [123]. The Cancer Genome Atlas (TCGA) performed unsupervised clustering of primary PC samples based on DNA methylation [123]. This analysis revealed an association of methylation patterns with the ETS fusion status was distinct on the basis of the ETS fusion partner (*ERG*, *ETV1*, *ETV4*, and *FLI1*) [123]. ERG fusion-positive tumors presented the most diverse methylation changes compared to the other groups [123]. In the same TCGA analysis, PC tumors with *SPOP, FOXA1,* and *IDH1* mutations also had distinct DNA methylation patterns. Interestingly, the IDH1-mutated PC subgroup had fewer DNA copy number alterations and was presented as an early onset PC subgroup. *IDH1* mutations have been associated with a DNA hypermethylator phenotype also in gliomas and hematological malignancies [123,124]. However, a study on early onset PC specimens did not report an association of methylation patterns with IDH1-mutated tumors [125]. In distinct early onset PCs, the same group developed a predictor of biochemical recurrence for these patients by integrating methylation patterns and gene expression, rather than DNA mutations [125]. The combination of gene expression and methylation pattern was also able to stratify patients with intermediate risk and Gleason score 7 [125].

DNA methylation in PC has also been studied in relation to changes in chromatin accessibility and histone modifications [72,106,111]. Kron et al. studied the association of DNA methylation of PCs with different disease grades and ERG status, and compared tumor methylation patterns to repressive (H3K27me3 and SUZ12) and to active (H3K4me3 and RNA pol II) chromatin profiles [111]. This analysis revealed that the differentially methylated regions (DMRs) were mostly hypermethylated in high Gleason score and ERG-positive tumors [111]. They also reported hypermethylated regions located near the gene *HOXD3* [111]. In their analysis, they found little overlap between DMRs associated with Gleason score and those that are associated with ERG fusion status, indicating different changes in DNA methylation in tumors with different disease grades and ERG status [111].

DNA methylation changes have been associated not only with PC carcinogenesis, but also with PC progression and treatment resistance. Promoter hypermethylation of *CRIP1*, *FLNC*, *RASGRF2*, *RUNX3*, and *HS3ST2* genes that are important for lineage commitment and differentiation have been associated with PC recurrence [105,126,127,128]. On the other hand, the promoter hypermethylation of particular genes, such as the *SRD5A2* gene that encodes for one of three isozymes of 5α-reductase, is significantly associated with longer survival in CRPC patients [129].

Peter et al. performed a genome-wide methylation analysis in cell lines representing PC progression and showed methylation changes associated with the development of CRPC and t-NEPC [130]. They showed that the development of ARSI treatment resistance leads to DNA hypermethylation but CRPC and t-NEPC models display similar numbers of hyper- and hypomethylated sites [130], suggesting that reconfiguration of DMRs might not be implicated in transdifferentiation between CRPC and NEPC.

In a large-scale study utilizing clinical specimens from 100 CRPC metastases, Zhao et al. showed that differential methylation preferably occurs at intergenic sites with regulatory functions [108]. Approximately 22% of tumors could be grouped into a CRPC subtype characterized by hypermethylation and somatic mutations in *TET2*, *DNMT3B*, *IDH1,* and *BRAF* genes [108]. They also showed that expression of PC driver genes, such as *AR*, *MYC,* and *ERG,* was associated with DNA methylation in regulatory intergenic regions [108].

We have profiled BPH, primary PC, and non-metastatic CRPCs, revealing that chromatin accessibility and DNA methylation are anticorrelated in these clinical specimens [19,72]. The majority of DMRs and differentially accessible regions between BPH, primary PC, and CRPCs tissue do not overlap, suggesting that the remodeling of accessible chromatin and DNA methylation in prostate carcinogenesis and progression to CRPC are two distinct mechanisms [72].

These studies show that DNA methylation patterns are altered during progression and development of treatment resistance, but additional studies are needed to further elucidate passenger and driver events linked to DNA methylation in PC. 

## 5. Dysregulation of Chromatin States through Histone Modification in Prostate Cancer

The organization and structure of the chromatin is the result of the activity of various proteins interacting with DNA. The negatively charged 2 nm DNA double helix wraps around positively charged histone octamers, creating the 10 nm fiber of adjacent nucleosomes. By continuous folding and looping, this primary structure is further compacted to higher order structures to form the 3D organization of the chromatin. Both DNA replication and RNA transcription require accessibility to DNA by reversible decondensation of these chromatin structures. Chromatin compaction and decondensation at certain loci are tightly regulated and cell type-specific. This is primarily achieved through the ability of chromatin-associated proteins to write (e.g., with acetyl transferase activity), read (e.g., with bromodomains), and erase (e.g., with demethylases) the histone code.

The histone code is the set of histone modifications (methylation, acetylation, etc.) defining the state of the chromatin. For instance, a chromatin region marked by methylation in lysine 4 of the histone 3 tail (typically marked as H3K4me1) and H3K36me1 could be defined as a promoter [131,132,133], but additional histone markers such as H3K27 Acetylation (H2K27Ac), H3K4me3, H3K27me3, or the presence of RNAPol2 could determine whether the promoter is actually active or poised [131,132,133,134]. ChromHMM is a tool that converts the histone code to an annotated chromatin state [133,134]. The presence of histone marks H3K4me1, H3K4me3, H3K27me3, H3K27Ac, H3K36me3, and H3K9me3 are used to annotate the chromatin state based on the Roadmap Epigenomics 18-state expanded model [135]. For example, in this model, active transcription is marked with H3K4me3, whereas repressed chromatin is marked with H3K27me3 and H3K9me3 heterochromatin marks [135]

**Figure 4:**
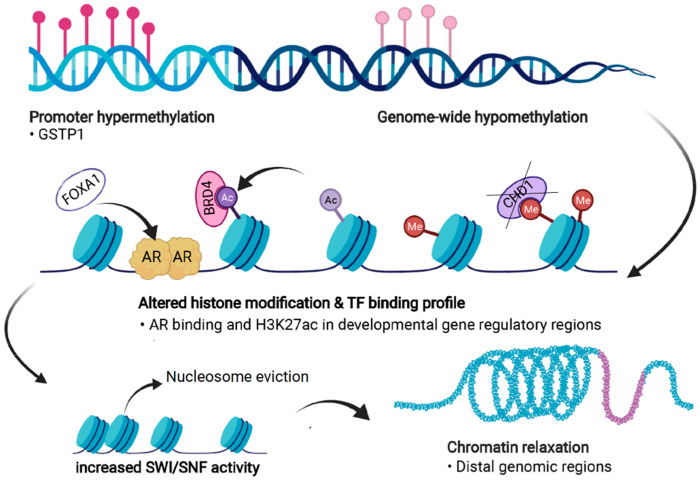
Forms of epigenetic and chromatin dysregulation in PC. DNA methylation is increased at specific regulatory regions, such as the *GSTP1* promoter, but is generally reduced genome-wide. Aberrant histone modifications especially at distal regulatory regions, often harboring binding motifs for key transcription factors (TFs) such as the androgen receptor (AR), shift to a more active state. This also occurs due to the action of transcriptional coactivators and chromatin readers such as BRD4, leading to recruitment of these TFs to previously repressed regions. Loss of *CHD1* induces chromatin rewiring, and pioneer factors such as FOXA1 are able to bind to repressed regions and recruit other TFs and histone remodeling complexes. Dysregulation of chromatin accessibility is partly due to increased activity of the SWI/SNF remodeling complex, which shifts the chromatin towards more permissive states, a process termed chromatin relaxation. Figure created with BioRender.com.

PC cells exhibit significant dysregulation of chromatin states (Figure 4). Firstly, androgen stimulation can trigger several changes in the chromatin states of PC cells. H3 acetylation has been demonstrated to accumulate at the *KLK3* (encoding PSA) promoter and enhancer regions upon androgen treatment in LNCaP cells [136,137,138]. In addition, the presence of androgens triggers the recruitment of histone acetylases (HATs) CBP and p300 to the regulatory regions of *KLK3*, thus changing the chromatin state to a more active one via their acetyl transferase activity [138,139]. p300 has also been shown to protect AR from degradation in PTEN-depleted PC mouse models, in which p300 was essential for the expression of AR target genes [140]. Moreover, combined inhibition of p300 and CBP significantly decreases the expression of programmed death-ligand 1 (PD-L1), which increases the efficacy of immunotherapy that aims to reactivate T-cells by PD-L1 blockade [141].

In addition to HATs, histone demethylases (JHDM2A, JMJD2C, and LSD1) have been reported to exhibit androgen stimulation-dependent recruitment to AR target genes such as *KLK3*, *TMPRSS2,* and *NKX3-1*, resulting in less methylated H3K9 and an active chromatin state [142,143,144]. Low levels of H3K9me2 have been shown to predict poor prognosis in PC patients [145], whereas higher IHC staining of H3K4me1 is associated with a higher risk of biochemical recurrence [146]. High levels of H3K4me2 are also associated with shorter time to biochemical recurrence after radical prostatectomy [147]. Despite active histone marks being associated with prognosis, suppressive chromatin states marked by H3K27me3 seem to be more abundant than active chromatin states marked by H3K4me3 in PC cells [148,149]. Ke et al. performed a genome-wide profiling of H3K27me3 and H3K4me3 in PC and normal epithelial cells and showed that while the number of each modification is relatively similar in both cells, the genome-wide profiles of these marks vary so that the genes marked by H3K27me3 or by both H3K27me3 and H3K4me3 are approximately 70% different [148]. Genes marked by H3K27me3 in PC cells are enriched with developmental functions, which was not the case for normal epithelial cells. This suggests that epigenetic reprogramming of developmental genes occurs during PC carcinogenesis. Examining genes that exhibited differential chromatin state in PC compared to normal epithelial cells, Ke et al. observed that frequent switches between H3K4me3 and H3K27me3 caused the strongest expression changes in genes, including most of the HOXA genes cluster [148].

Changes in chromatin states have also been associated with PC progression. The expression of the aforementioned HATs p300 and CBP was shown to be significantly higher in metastatic CRPC lesions than in local tumors [150]. p300 functions to stabilize a histone demethylase JMJD1A, which is upregulated in CRPC in comparison to PC, increasing JMJD1A recruitment to AR target genes and thus, facilitating the conversion of the chromatin state into an active one [151]. The levels of H3K4me1, H3K4me2, and H3K4me3 based on IHC increase during PC progression [146]. This is interesting as the binding of FOXA1 to its targets strongly depends on the distribution of H3K4me1 and H3K4me2 [93,152]. Selective accumulation of these histone marks is found at the AR-bound enhancers of M-phase cell cycle genes, such as *CDK1* and *UBEC2*, in CRPC tissues and cell models when compared to androgen-dependent cell lines and tumor tissue samples [153]. Consistently analyzing global levels of H3K27me3 by IHC, Pellakuru et al. showed that the levels decrease as markers of aggressive disease increase [154]. This decline in H3K27me3 levels is also observed when more differentiated normal prostatic luminal epithelial cells are compared to more stem-like basal cells [154].

These findings suggest that overall active chromatin state increases during PC progression and that the epigenome is reprogrammed to resemble stem-like cells. Supporting this, genes repressed and marked by H3K27me3 levels in normal prostate tissue and derepressed during PC progression are developmental regulators and homeobox proteins [155,156]. Yu et al. revealed that such epigenetic reprogramming might partly originate from the aberrant function of EZH2 [155,156]. Many studies have now shown that EZH2 is overexpressed in PC and even more so in CRPC [157,158,159,160]. However, it has been shown to also have a PRC2-independent role in CRPC [161]. Mechanistically, EZH2 functions as a co-activator for important transcription factors such as AR upon phosphorylation [161], and as a part of the PRC2 complex to induce silenced chromatin states.

The combination of aberrant functions of HATs, demethylases, and EZH2 all seem to contribute to increased active chromatin states in PC progression and treatment resistance. In line with this, Pomerantz et al. showed that the profiles of active enhancers defined by H3K27ac are similar in CRPC and prostate-specific fetal tissue, implicating reactivation of the developmental transcriptional programs in late-stage disease [64].

## 6. Dysregulation of Chromatin Accessibility

Chromatin accessibility is facilitated by the chromatin remodeling complexes by either ejecting histones or full nucleosomes or by sliding nucleosomes to different positions in the DNA [162,163]. Several publications have linked the chromatin remodeling complex SWI/SNF to PC carcinogenesis and progression. BRG1 (also known as SMARCA4), a core subunit of the SWI/SNF complex, is required for chromatin accessibility [163]. Previous studies have reported the increased expression of BRG1 in primary PC [164,165,166,167]. Higher BRG1 expression has been associated with larger tumor mass and increased invasion of PC-3 cells [166]. Inhibition of BRG1 in PC-3 cell lines has also been demonstrated to decrease cell proliferation and induce apoptosis [168]. Inhibition of BRG1 in mouse xenografts increased the survival of the mice and decreased tumor growth [168]. Moreover, its increased expression has been associated with more malignant features [164,166,167,168]. Expression of BRG1 has a positive correlation with Gleason score, as it increases as the tumor progresses and the highest expression is observed in NEPC [164,166,167,168].

PTEN loss is associated with PC progression to CRPC [169] and has been demonstrated to stabilize, and thus, increase, the amount of BRG1 protein [167]. IHC staining of radical prostatectomy tissues demonstrated that in tumors exhibiting low PTEN expression, high BRG1 expression correlated with poor outcome [167]. PTEN and BRG1 were indeed shown to be synthetic lethal in a panel of PC cell lines and in a mouse model [167]. Open chromatin regions were reduced by approximately 60% when BRG1 was depleted in cells lacking PTEN, highlighting the importance of this SWI/SNF subunit to chromatin accessibility [167].

In addition to BRG1, increased expression of SWI/SNF complex subunits BAF57 and BAF155 has been described in PC [170,171,172]. Inhibition of BAF57 results in the inhibition of AR-dependent genes in PC cell lines [172]. IHC studies of primary tumors and metastatic specimens have revealed that BAF57 expression positively correlates with Gleason score [170,172]. Moreover, cell line studies with LNCaP and C4-2 (a CRPC cell line) showed that BAF57 mediates ligand-independent AR activity, thus inducing the expression of migration- and metastasis-related genes in the absence of androgens [170]. TMA IHC analysis of BAF155 revealed the highest staining in recurrent and metastatic prostate cancer specimens and a positive correlation with Gleason score [171].

Interestingly, the differential expression of certain SWI/SNF complex subunits has been linked to the progression of PC into NEPC [164]. For example, the RNA and protein expression of BRG1 and neuron-specific SWI/SNF subunit proteins, such as BAF53B and BAF45B, exhibited significantly higher expression in NEPC than in CRPC [164]. This suggests that differential chromatin remodeling is required for the progression of prostate cancer to CRPC and NEPC.

Abnormal activation of chromatin-related proteins such as chromatin readers and the SWI/SNF complex and its subunits suggests an overall increase in accessible chromatin in gene regulatory regions in CRPC. Indeed, multiple studies have shown that relaxed chromatin correlates with PC progression [19,72,145,154,173,174] and that different markers for open chromatin can function as predictors for tumor recurrence [175,176]. Moreover, Bromodomain and Extraterminal domain (BET) proteins BRD2 and BRD4, chromatin readers that recognize acetylated histones and function to promote transcription, have been shown to maintain accessible chromatin in PC [19,177,178,179] and to be overexpressed in CRPC [19]. These proteins have been demonstrated to mediate tumor growth in both CRPC cell lines and in vivo experiments with xenograft models [180,181,182,183]. In addition, BET proteins are associated with the increased expression of migration- and invasion-related genes in CRPC [184,185]. Although BRD4 can co-regulate invasion-related genes together with ERG [184], it seems that the main mechanism of function for these proteins is to enhance AR-mediated transcriptional regulation [180,181,182,183,186], highlighted by the fact that AR-negative cells are not sensitive to BET inhibitors [180]. AR overexpression has been associated with the chromatin accessibility maintenance activities of BET proteins BRD4 and BRD2 together with AR-regulated bromodomain-containing protein ATAD2 [19], indicating that these proteins remodel chromatin to promote AR-mediated gene regulation.

Similarly, the SWI/SNF complex has been shown to be an important transcriptional cofactor for AR activity [172,187,188,189,190]. Multiple studies have shown that SWI/SNF activity is critical for the AR-mediated activation of *KLK3* and *TMPRSS2* transcription [172,188,190]. In addition, SWI/SNF activity positively regulates the expression of other androgen regulated genes, such as *FKBP5* and *KLK2* [188]. Inhibition of different subunits of the SWI/SNF complex inhibits the proliferation of AR-dependent PC cells [188,189], but different subunits of the SWI/SNF complex seem to promote AR-mediated activation on different target genes [190]. This highlights the dynamic nature of SWI/SNF function in mediating transcriptional plasticity in response to potential PC treatments.

The importance of bromodomain activity in modifying the structure of the chromatin in PC has been reviewed previously [191]. Here, we add that Welti et al. recently reported that targeting p300/CBP, histone acetyltransferases with known AR transcriptional coactivator function, could be a new therapeutic strategy for CRPC, and describe a novel small-molecule inhibitor (CCS1477) of the p300/CBP conserved bromodomain [150].

These findings demonstrate the abnormal function of chromatin accessibility regulators in PC and in the progression to treatment resistance. These changes precede the reprogramming of TF chromatin binding, especially the binding of the AR.

## 7. Dysregulation of Chromatin Accessibility and Regulation of Transcription Factors Binding to DNA

There is an evident interplay between chromatin accessibility, chromatin remodelers, and TF activity in promoting prostate carcinogenesis and progression to CRPC by mediating transcriptional plasticity. Active chromatin is generally open and accessible, whereas repressed chromatin does not allow access to DNA [192]. Excluding so-called pioneering factors such as FOXA1 that can bind to closed chromatin and recruit chromatin remodeling machinery, most TFs bind to chromatin that is already in open conformation [192]. On the other hand, repressive epigenetic marks such as DNA methylation correlate negatively with open chromatin regions [19,72,193]. Chromatin accessibility, especially at the distal (non-promoter) intergenic regions, strongly reflects the role of epigenetics in the definition of cellular identity (lineage commitment and plasticity), as normal or cancer cell type-specific open regions mostly reside in these regions [192,193]. These regions contain enhancers, which are normally the most prominent tissue-specific gene regulatory regions [194]. Motif analysis of accessible chromatin regions can be used as a readout for TF binding [195]. This information is useful for interpreting which factors are likely responsible for the tissue- or disease stage-specific regulation [195]. We studied accessibility between BPH, primary PC, and non-metastatic CRPC, which revealed that the promoters of expressed genes mostly exhibit accessible chromatin across all sample types, whereas other genomic regions, such as enhancers, exhibit specific accessibility patterns linked to disease stage but also display high heterogeneity between individual tumors [72]. Overall, we found that the chromatin shifts towards more accessible states during progression to CRPC. TF footprinting of accessible DNA regions indicated that FOXA1, AR, and HOXB13 binding sites have a higher accessibility in PC specimens, which is further reprogrammed during progression to CRPC [72]. This supports previous findings that the AR cistrome undergoes extensive rewiring and expansion during prostate carcinogenesis and emergence of therapy resistance [61,64,196]. Therefore, we suggested that the reprogramming of the AR cistrome might be the result of an altered chromatin structure that remodels the repertoire of possible binding sites for AR [197]. This is consistent with enhanced chromatin binding of AR itself in AR-overexpressing PC tumors [198], as well as the reprogrammed chromatin binding of other TFs during prostate carcinogenesis and progression.

In addition to AR, its coregulators have been extensively studied during PC progression [199]. AR cofactors such as the ETS gene family of TFs, including ERG, are often overexpressed in PC due to recurrent somatic translocations [200]. The ETS family of TFs has been shown to extensively reprogram the binding of AR to the chromatin [201,202,203,204], leading to obstruction of epithelial differentiation [201,202]. Mechanistically, this would occur through blocking AR binding at select sites via recruitment of EZH2 and histone deacetylases, and recruitment of AR at new sites [201,202]. The binding of AR, in this case, occurs at primed enhancer regions (characterized by H3K4me1), as guided by ERG [201,202,203]. On the other hand, ERG can induce enhancer activation (marked by H3K27Ac) in new regions, accompanied by changes in the binding profiles of other transcriptional master regulators such as FOXA1 and HOXB13 [204]. However, Li et al. showed that ERG is also responsible for maintaining luminal differentiation and suppressing basal differentiation of PC cells in a mouse model [205]. This dual role of ERG suggests that functions of transcriptional master regulators in PC are highly context-dependent.

The proportion of ETS fusion-positive tumors does not increase during progression of PC to CRPC [88], indicating that these fusions and their associated regulatory changes might contribute to PC development but are not necessarily involved in PC progression. The possible role of ETS fusions in prostate carcinogenesis is confirmed by the fact that early onset PCs have a higher proportion of *TMPRSS2-ERG* fusions than late onset PCs, in addition to these tumors being generally more aggressive [125,206].

The AR cistrome expands during PC development and is further reprogrammed as the cancer progresses to CRPC, as shown by Sharma et al. and Pomerantz et al. [61,63,64]. This reprogramming of the AR cistrome reactivates the regulatory regions that control the expression of genes involved in prostate development [64], suggesting that dedifferentiation to a more stem-like state could be a common mechanism by which cells overcome the effects of treatment, as discussed also in the previous chapters. The AR binding sites that are reactivated in progression to CRPC are already occupied by FOXA1 and HOXB13 during primary PC development, but AR binding is not present despite these chromatin regions being open [64]. This suggests that additional unknown chromatin factors may prevent AR binding to these sites in primary PC. Using PC cell lines, we showed that overexpression of c-MYC, a phenomenon that occurs in about 30% of CRPCs [207], interferes with the AR transcriptional program [208]. Recent evidence in in vivo models reported by Qiu et al. showed that this effect might be mediated by the alteration of RNA POL2 recruitment at active chromatin sites [209], which is consistent with chromatin conformation changes. Recently, He et al. showed that in CRPC cell lines resistant to enzalutamide, AR is recruited to the chromatin, even in regions lacking canonical androgen-responsive elements [210]. Using both bulk and single cell chromatin accessibility data in models of enzalutamide resistance, we have recently shown that enzalutamide resistance is associated with chromatin relaxation and reprogrammed accessibility [211], which is consistent with potential alterations of TF binding in these cells, including the reprogramming of AR binding.

He et al. showed that AR binding converges toward the regulatory regions of genes such as *ID1*, *ID3*, and *PFN2*, which are androgen response element-independent AR target genes essential for CRPC growth in enzalutamide-resistant CRPC cells, patient-derived xenografts, and tissue samples [210]. The expression of these genes is also associated with poor survival in enzalutamide-treated patients [210]. Interestingly, chromatin immunoprecipitation experiments revealed that CXXC5, which can induce changes in chromatin state [212], and TET2, which promotes DNA demethylation [213], mediated such binding events at unmethylated CpG islands, revealing a potential role for these TFs in enzalutamide resistance [210] and linking chromatin relaxation to differential DNA methylation, differentiation, and lineage commitment. The gained AR binding sites as a consequence of cooperative activity with CXXC5 and TET2 were enriched with H3K27Ac, and treatment-resistant cells, organoids, and patient-derived xenografts were shown to exhibit hypersensitivity to the dual inhibition of BET and CBP/p300 proteins [210], highlighting the importance of chromatin states in the emergence of treatment resistance. Given the heterogeneity of PC, it is plausible that other TFs may become activated and play secondary roles in transcriptional reconfiguration during the emergence of treatment resistance [64]. Examples of such TFs could be the pluripotent stem cell TF SOX2, which was found to drive development of the prostate [214], and RUNX1, which was shown to characterize regenerative cells in CRPC [215]. Expression and activity of SOX2 and GR have been shown to promote enzalutamide resistance [12,13,14,15]. GR dependency in CRPC enzalutamide-resistant models was demonstrated to be mediated by bromodomain activity [216]. Exposure to enzalutamide can also alter the activity of other chromatin regulators [6]. n-Myc–mediated epigenetic reprogramming has been shown to drive lineage plasticity in CRPC [82], and the induction of core pluripotency master regulators in PC cells has been associated with poor clinical outcome and treatment resistance [217].

These findings suggest that chromatin reprogramming can lead to the recruitment of additional TFs during PC progression to CRPC.

## 8. Chromatin Conformation and 3D Disorganization

In addition to nucleosome remodeling, chromatin accessibility, and chromatin state, the higher order chromatin organization influences gene expression [218]. Although the looping of chromatin induces the organization of TADs and not the other way around, formed TADs do, to some extent, constrict the movement of chromatin loops and result in a higher probability of gene regulatory region looping within a TAD [219]. Therefore, both the loop structures and the TADs have a functional role [218]. Loops facilitate physical interactions between enhancers and promoters, whereas TADs assist their interactions by bringing them close together in 3D space [218]. Epigenetic modifications can also function as docking sites for regulatory and structural proteins anchoring the chromatin 3D structure (e.g., lamin, CTCF, and cohesin).

Multiple chromosome conformation capture sequencing studies from cell lines have demonstrated that the 3D organization of the PC genome is reprogrammed in comparison to normal cells [220,221,222]. The general interactome remains unaltered, but at least in PC cell lines, TADs normally found in normal cells are split into smaller cancer-specific TADs [220,221,222]. The boundaries of the newly formed cancer-specific TADs are enriched in regions that display copy number variation. For example, a common deletion of the 17p13.1 locus splits a TAD found in normal cells into two PC-specific TADs, indicating that structural changes in the PC genome partly induce changes in functional gene regulatory networks [220,221]. Moreover, newly formed cancer-specific TADs were shown to be comparable between different PC cell lines, indicating that TAD reorganization is cancer-specific rather than a stochastic process [220,221]. ChIP-seq studies have indicated that TAD reorganization may function to promote the expression of PC-related genes. Taberlay et al. observed an enrichment in CTCF-bound regions, enhancers, and promoters in cancer-specific TADs [221].

Rhie et al. used normal and PC cell lines to study the association of TADs with different chromatin states [222]. They showed that several TADs change when comparing normal cells to PC cells. They also noted that although there are many TADs that are unaltered, the chromatin structure of these regions undergoes dramatic changes [222]. Heterochromatin TADs that are marked with H3K9me3 are common to both normal and cancer cells and undergo the most changes, with 66.5% having a different chromatin state in cancer cells compared to normal cells. TADs containing H3K27me3 and H3K36me3 exhibit fewer changes between normal and cancer cells [222]. Within TADs that maintain the same TAD boundaries but exhibit a change in chromatin state, 1185 genes were upregulated and 713 downregulated in cancer cells, suggesting that chromatin states become slightly more open and active in cancer cells within these TADs [222]. This is in line with the above-discussed evidence, pointing towards increasing chromatin accessibility during PC development and progression. Furthermore, several genes with higher expression in PC were looped to cancer-specific active enhancers and included binding sites for PC signature TFs, such as FOXA1 and AR [222]. FOXA1-bound enhancers were additionally shown to be looped to specific, alternative promoters in PC compared to normal cells, indicating that chromatin reorganization drives both FOXA1 expression and its binding to target genes through enhancer–promoter loops [222].

The functional role of chromatin loops in the AR cistrome was further evaluated in a meta-analysis of protein–chromatin interactions (AR, FOXA1, and CTCF) and active enhancer regions in PC cells [222]. The results indicated that the loops that contain either AR or FOXA1 binding sites are focused on genes expressed highly in prostate tissue and that these genes are also enriched in PC [223]. Knockdown of CTCF, with consequent abolishment of many chromatin loops, significantly downregulated the expression of AR-responsive genes [223]. This indicates that loop structures may function to promote AR-dependent gene expression and thus, changes in loop structures might participate in the expansion of the AR cistrome.

The pioneering factor FOXA1, together with the mediator complex component MED1, were first shown to facilitate chromatin looping at the *UBE2C* locus in CRPC models [224]. *UBE2C* has been shown by several groups to mediate castration-resistance [153,225,226,227,228,229], further highlighting the importance of chromatin loops in gene expression and PC progression.

Baca et al. showed that some regulatory regions gain active histone marks in NEPC in comparison to PC and that these regions form more physical contacts with genes exhibiting higher expression in NEPC than in PC [77]. Thus, the loop structures are likely to change together with transcription to promote lineage plasticity [77]. This evidence suggest that chromatin loops are highly dynamic and reprogrammed during PC progression and might have a crucial role in the reprogramming of the activity of PC-specific TFs [219].

Genomic rearrangements and breakpoints in PC have been shown to occur commonly at sites that are annotated as active enhancers in accessible chromatin regions or at sites that are transcriptionally active in PC datasets [125]. A similar association with genomic breakpoints was also found at AR binding sites [206]. This seems to exhibit higher specificity in early onset PCs than in late onset PCs [125], suggesting that the chromatin of late onset PCs is more relaxed than that of early onset PCs. Consistently, Hi-C analysis of chromatin loops and H3K27Ac (an active enhancer mark) significantly correlated with the number of breakpoints in early onset but not in late onset PC.

Predisposing risk SNPs have also been shown to exhibit aberrant chromatin looping [230,231,232,233]. Cell line studies of 10 risk loci SNPs residing in non-coding regions revealed that there are significant interactions between the risk loci and their potential target genes, and these interactions often co-localize with active histone marks [230]. Zhang et al. demonstrated that AR and ERG create long-range chromatin interactions and their co-binding sites are significantly enriched by risk SNPs in PC cells. These TFs act to establish chromatin interactions that regulate a subset of AR target genes important for the promotion of PC tumor growth [234]. The chromatin loops involved may also function to link androgen upregulated lncRNAs to their target genes [234]. For example, three clinically relevant lncRNAs (*PCAT43*, *PCAT61*, and *PCAT76*) in the *PMEPA1* gene locus are connected to the gene by a chromatin loop facilitated by AR and ERG. Depletion of the lncRNAs *PCAT43* and *PCAT61* significantly decreases the AR-mediated activation of *PMEPA1*, indicating that lncRNAs may function to converge some AR-mediated responses via established chromatin loops [234].

Analysis of the hotspot mutation locus 8q24, which is located in an enhancer and associated with increased PC risk, revealed that it is connected via chromatin loops to several oncogenes and is responsible for their regulation [231]. Pioneer factors and key PC-specific TFs, such as FOXA1 and AR, were shown to co-localize to these interactomes [231]. In addition, the 7p15.2 locus forms a long-range chromatin interaction with the *HOXA* locus, repressing the expression of HOXA13 [232]. Deletion of this locus leads to loss of *HOXA13* suppression and its overexpression, which, in normal immortalized RWPE-1 cells, has been found to induce the activation of the HIPPO pathway [232]. The activation of the HIPPO pathway has been suggested to promote castration-resistance [235,236]. Thus, deregulation of *HOXA13* via the loss of a suppressive chromatin loop may be implicated in promoting prostate carcinogenesis and tumor progression.

In summary, although chromatin relaxation during PC progression to CRPC particularly occurs in enhancers as mentioned in earlier sections [19,72], a state of relaxed chromatin has been observed in late stage disease that does not necessarily overlap with gene regulatory regions [197]. In addition to affecting the transcriptional output in malignant cells, chromatin relaxation is associated with breakpoints of genomic rearrangements at open chromatin regions, alterations of chromatin looping, and 3D structure organization of the chromatin, thereby contributing to oncogene activation.

## 9. Clinical Aspects and Future Directions

A thorough characterization of the processes leading to epigenetic and chromatin dysregulation will allow for a more refined classification of PC subtypes and provide rationale for novel therapeutic approaches for both HSPC and CRPC patients.

Although genes with chromatin regulatory functions are known to be mutated and altered in terms of their expression in many cancer types, further studies are needed to understand the mechanisms by which the alteration of these genes leads to chromatin dysregulation and carcinogenesis. Recently, Yuan et al. [237] and Li et al. [238] demonstrated that the oncogenic transformation of the *NSD3* gene due to overexpression or missense mutation leads to the increased expression of other oncogenes via H3K36 dimethylation. This makes the NSD gene family an attractive therapeutic target. Similarly, Grbesa et al. showed that *SPOP* mutations increase chromatin accessibility in regions that coincide with AR binding sites in primary tumors and confer exquisite sensitivity to AR-targeted therapies due to the interplay of AR with the chromatin [62]. Conversely, cancer therapies such as radiotherapy may lead to epigenetic changes that drive cancer progression, as discussed by Cabrera-Licona et al. [239].

Another aspect that deserves further investigation is the presence of conserved shadow enhancers in the genome, which are alternative, redundant enhancers for the regulation of particular genes [240]. The use of these enhancers may contribute to gene expression alterations, particularly when permissive chromatin in a relaxed state is reprogrammed and increases in quantity during PC progression. Future studies should, therefore, investigate the contribution of the utilization of these enhancers to gene expression dysregulation.

To date, epigenetic studies of PC have mostly been performed by bulk sequencing of individual tumors or in cell lines, and the full heterogeneity of PC phenotypes might still be severely underexplored. Single cell-based studies might be able to shed light on this complexity by increasing the analytical resolution and allowing for analysis of the transcriptome, chromatin structure, and epigenetics in the same cells.

In addition to DNA modifications, links are currently being explored between PC and epitranscriptomics such as mRNA methylation. N^6^-methyladenosine (m^6^A) is the most common post-transcriptional modification of mRNA, and its dysregulation via the *METTL3* gene has been shown to play a role in prostate carcinogenesis and PC progression through modulation of the Wnt pathway [241] and *MYC* mRNA methylation [242]. METTL3 also regulates the expression of Integrin β1, which may facilitate PC metastasis to bone [243].

Additionally, protein modifications can have further effects on PC biology. This is exemplified by the O-GlcNAc modification of proteins that are upregulated in early stage PC [244]. Recent work has shown that inhibiting O-GlcNAc transferase in conjunction with CDK9 is lethal to organoid cells from CRPC patients [245]. Interestingly, O-GlCNacylation of the chromatin has also been described in PC [246], but its role in gene regulation and contribution to prostate carcinogenesis and progression to CRPC need to be studied further.

Limited focus on only cancer cells might also be too narrow a scope to understand the full complexity of the epigenetic landscape of prostate tumors. In PC, the TME consists of stromal cells such as smooth muscle, fibroblasts, endothelial cells, and leukocytes. The interplay of PC cells with the TME in resistance to ARSI has been revealed in recent single cell studies by Chen et al. and He et al. [71,247]. These studies showed how tumor cells can modulate the TME, including resident immune cells and the stromal endothelial cells, during cancer progression and emergence of therapy resistance [71,247]. These modulatory effects are apt to suppress the host immune response, as well as create pre-metastatic niches in the sentinel lymph nodes and potentially other metastatic sites [71,247]. On the other hand, the TME has been shown to affect PC cells and contribute to ARSI resistance. For example, Zhang et al. recently showed that cancer associated fibroblasts (CAFs) can confer ARSI resistance by upregulating NRG1, the ligand of ERBB3, which leads to activation of the PI3K-AKT pathway [248]. As ERBB3 inhibition with a blocking antibody under development (GSK2849330) has been effective in NRG fusion-positive squamous cell carcinoma of the lung [249], it might be beneficial in CRPCs driven by CAF-mediated activation of ERBB3 [248]. CAFs have been previously shown to induce chemotherapy resistance by secreting WNT16B and activating B-catenin signaling in the tumor cells [250]. Although the effects of epigenetic plasticity need to be further investigated in the context of the interplaying mechanisms between PC and the TME, these effects are likely to impact TF activity hijacking, as well as chromatin and transcriptional reprogramming [196]. However, the mechanisms by which, for example, the TME regulates chromatin structure of the tumor cells via altered paracrine signaling or by altering their metabolic state, are still poorly understood. Further research would help to clarify aspects such as the influence of lipid composition and high fat diet in PC emergence, tumor aggressiveness, and resistance to ARSI [251,252].

Finally, given the importance of chromatin and epigenetic dysregulation in prostate carcinogenesis and PC progression to CRPC, many investigators have trialed and continue to trial so-called epidrugs and their efficacy in different clinical settings. A comprehensive review of the ongoing clinical trials testing small molecules in PC has been recently published by Kumaraswamy et al. [253]. Here, we emphasize that since most of these trials still do not have companion biomarkers, more research is needed to provide clear indications of the utility of certain classes of small molecules in the highlighted PC subgroups. For instance, the DNA methylation levels of the *GSTP1* promoter were already suggested as a biomarker in the 1990s [103], but despite all the knowledge and studies so far on DNA methylation, we still lack methylation-based biomarkers. The use of RNA-based signatures from tissue samples as a surrogate of deregulated chromatin footprints could be a strategy to provide such indications [254]. As an example, we previously developed BROMO-10, a molecular classifier to prognosticate progression of PC under ADT, development of metastasis, and predict response to BET inhibitors [19]. Other molecular signatures are also being developed [254], including biomarkers from liquid biopsies [255]. The challenge ahead is to prove the use of these classifiers in the clinical setting [256].

## 10. Conclusions

Chromatin- and epigenetics-related processes contribute to prostate carcinogenesis and prostate cancer progression to castration-resistance under selective treatment pressure. Prostate cancer shows evidence of progressive chromatin relaxation, reconfiguration of the 3D chromatin structure, alteration of specific histone modifications, and methylation patterns that contribute to so-called transcriptional plasticity and ultimately, provide the means of cancer cell adaptation to treatment. A total of 5% and 11% of genes coding for chromatin-associated proteins are recurrently mutated or aberrantly expressed in primary PC and in CRPC compared to the normal prostate tissue, respectively. In particular, recurrently mutated chromatin modifiers are highly expressed in CRPC, irrespective of their mutation frequency. Moreover, 6% of genes coding for chromatin-associated proteins are overexpressed during prostate carcinogenesis and treatment resistance. Targeting epigenetics- and chromatin-related processes in combination with existing therapeutic approaches seems to be an attractive strategy for providing greater benefits to prostate cancer patients. However, a deeper understanding of the underlying mechanisms involved and better biomarkers are needed to implement this strategy in the clinic.

## Supplementary Materials

The following are available online at https://www.mdpi.com/article/10.3390/cancers13133325/s1, Table S1: Surface and transcriptional markers of club-like cells in the prostate in recent single cell analyses. Table S2: Mutation frequencies of genes coding for chromatin-associated proteins in the ICGC prostate adenocarcinoma cohorts and castration-resistant prostate cancer (CRPC) cohorts.

## References

[1] Siegel R.L., Miller K.D., Fuchs H.E., Jemal A. (2021). Cancer Statistics, 2021. CA Cancer J. Clin..

[2] Sathianathen N.J., Koschel S., Thangasamy I.A., Teh J., Alghazo O., Butcher G., Howard H., Kapoor J., Lawrentschuk N., Siva S. (2020). Indirect Comparisons of Efficacy between Combination Approaches in Metastatic Hormone-Sensitive Prostate Cancer: A Systematic Review and Network Meta-Analysis. Eur. Urol..

[3] Heidenreich A., Bastian P.J., Bellmunt J., Bolla M., Joniau S., van der Kwast T., Mason M., Matveev V., Wiegel T., Zattoni F. (2014). EAU Guidelines on Prostate Cancer. Part II: Treatment of Advanced, Relapsing, and Castration-Resistant Prostate Cancer. Eur. Urol..

[4] Tosco L., Briganti A., D’amico A.V., Eastham J., Eisenberger M., Gleave M., Haustermans K., Logothetis C.J., Saad F., Sweeney C. (2019). Systematic Review of Systemic Therapies and Therapeutic Combinations with Local Treatments for High-Risk Localized Prostate Cancer. Eur. Urol..

[5] Moris L., Cumberbatch M.G., Van den Broeck T., Gandaglia G., Fossati N., Kelly B., Pal R., Briers E., Cornford P., De Santis M. (2020). Benefits and Risks of Primary Treatments for High-Risk Localized and Locally Advanced Prostate Cancer: An International Multidisciplinary Systematic Review. Eur. Urol..

[6] Beltran H., Prandi D., Mosquera J.M., Benelli M., Puca L., Cyrta J., Marotz C., Giannopoulou E., Chakravarthi B.V.S.K., Varambally S. (2016). Divergent Clonal Evolution of Castration-Resistant Neuroendocrine Prostate Cancer. Nat. Med..

[7] Formaggio N., Rubin M.A., Theurillat J.-P. (2021). Loss and Revival of Androgen Receptor Signaling in Advanced Prostate Cancer. Oncogene.

[8] Watson P.A., Arora V.K., Sawyers C.L. (2015). Emerging Mechanisms of Resistance to Androgen Receptor Inhibitors in Prostate Cancer. Nat. Rev. Cancer.

[9] Beltran H., Tomlins S., Aparicio A., Arora V., Rickman D., Ayala G., Huang J., True L., Gleave M.E., Soule H. (2014). Aggressive Variants of Castration-Resistant Prostate Cancer. Clin. Cancer Res..

[10] Handle F., Prekovic S., Helsen C., Van den Broeck T., Smeets E., Moris L., Eerlings R., El Kharraz S., Urbanucci A., Mills I.G. (2019). Drivers of AR Indifferent Anti-Androgen Resistance in Prostate Cancer Cells. Sci. Rep..

[11] Bluemn E.G., Coleman I.M., Lucas J.M., Coleman R.T., Hernandez-Lopez S., Tharakan R., Bianchi-Frias D., Dumpit R.F., Kaipainen A., Corella A.N. (2017). Androgen Receptor Pathway-Independent Prostate Cancer Is Sustained through FGF Signaling. Cancer Cell.

[12] Crona D.J., Whang Y.E. (2017). Androgen Receptor-Dependent and -Independent Mechanisms Involved in Prostate Cancer Therapy Resistance. Cancers.

[13] Isikbay M., Otto K., Kregel S., Kach J., Cai Y., Vander Griend D.J., Conzen S.D., Szmulewitz R.Z. (2014). Glucocorticoid Receptor Activity Contributes to Resistance to Androgen-Targeted Therapy in Prostate Cancer. Horm. Cancer.

[14] Li J., Alyamani M., Zhang A., Chang K.-H., Berk M., Li Z., Zhu Z., Petro M., Magi-Galluzzi C., Taplin M.-E. (2017). Aberrant Corticosteroid Metabolism in Tumor Cells Enables GR Takeover in Enzalutamide Resistant Prostate Cancer. eLife.

[15] Mu P., Zhang Z., Benelli M., Karthaus W.R., Hoover E., Chen C.-C., Wongvipat J., Ku S.-Y., Gao D., Cao Z. (2017). SOX2 Promotes Lineage Plasticity and Antiandrogen Resistance in TP53- and RB1-Deficient Prostate Cancer. Science.

[16] Braadland P.R., Ramberg H., Grytli H.H., Urbanucci A., Nielsen H.K., Guldvik I.J., Engedal A., Ketola K., Wang W., Svindland A. (2019). The β2-Adrenergic Receptor Is a Molecular Switch for Neuroendocrine Transdifferentiation of Prostate Cancer Cells. Mol. Cancer Res..

[17] Jia L., Shen H.C., Wantroba M., Khalid O., Liang G., Wang Q., Gentzschein E., Pinski J.K., Stanczyk F.Z., Jones P.A. (2006). Locus-Wide Chromatin Remodeling and Enhanced Androgen Receptor-Mediated Transcription in Recurrent Prostate Tumor Cells. Mol. Cell. Biol..

[18] Tewari A.K., Yardimci G.G., Shibata Y., Sheffield N.C., Song L., Taylor B.S., Georgiev S.G., Coetzee G.A., Ohler U., Furey T.S. (2012). Chromatin Accessibility Reveals Insights into Androgen Receptor Activation and Transcriptional Specificity. Genome Biol..

[19] Urbanucci A., Barfeld S.J., Kytölä V., Itkonen H.M., Coleman I.M., Vodák D., Sjöblom L., Sheng X., Tolonen T., Minner S. (2017). Androgen Receptor Deregulation Drives Bromodomain-Mediated Chromatin Alterations in Prostate Cancer. Cell Rep..

[20] Brücher B.L.D.M., Jamall I.S. (2016). Somatic Mutation Theory—Why It’s Wrong for Most Cancers. Cell. Physiol. Biochem..

[21] The ICGC/TCGA Pan-Cancer Analysis of Whole Genomes Consortium (2020). Pan-Cancer Analysis of Whole Genomes. Nature.

[22] Barbieri C.E., Baca S.C., Lawrence M.S., Demichelis F., Blattner M., Theurillat J.-P., White T.A., Stojanov P., Van Allen E., Stransky N. (2012). Exome Sequencing Identifies Recurrent SPOP, FOXA1 and MED12 Mutations in Prostate Cancer. Nat. Genet..

[23] Hanahan D., Weinberg R.A. (2000). The Hallmarks of Cancer. Cell.

[24] Hanahan D., Weinberg R.A. (2011). Hallmarks of Cancer: The next Generation. Cell.

[25] Flavahan W.A., Gaskell E., Bernstein B.E. (2017). Epigenetic Plasticity and the Hallmarks of Cancer. Science.

[26] Darwiche N. (2020). Epigenetic Mechanisms and the Hallmarks of Cancer: An Intimate Affair. Am. J. Cancer Res..

[27] Mikkelsen T.S., Ku M., Jaffe D.B., Issac B., Lieberman E., Giannoukos G., Alvarez P., Brockman W., Kim T.-K., Koche R.P. (2007). Genome-Wide Maps of Chromatin State in Pluripotent and Lineage-Committed Cells. Nature.

[28] Rubin M.A., Bristow R.G., Thienger P.D., Dive C., Imielinski M. (2020). Impact of Lineage Plasticity to and from a Neuroendocrine Phenotype on Progression and Response in Prostate and Lung Cancers. Mol. Cell.

[29] Brocks D., Assenov Y., Minner S., Bogatyrova O., Simon R., Koop C., Oakes C., Zucknick M., Lipka D.B., Weischenfeldt J. (2014). Intratumor DNA Methylation Heterogeneity Reflects Clonal Evolution in Aggressive Prostate Cancer. Cell Rep..

[30] Fontugne J., Davis K., Palanisamy N., Udager A., Mehra R., McDaniel A.S., Siddiqui J., Rubin M.A., Mosquera J.M., Tomlins S.A. (2016). Clonal Evaluation of Prostate Cancer Foci in Biopsies with Discontinuous Tumor Involvement by Dual ERG/SPINK1 Immunohistochemistry. Mod. Pathol..

[31] Salami S.S., Hovelson D.H., Kaplan J.B., Mathieu R., Udager A.M., Curci N.E., Lee M., Plouffe K.R., de la Vega L.L., Susani M. (2018). Transcriptomic Heterogeneity in Multifocal Prostate Cancer. JCI Insight.

[32] Talos F., Mitrofanova A., Bergren S.K., Califano A., Shen M.M. (2017). A Computational Systems Approach Identifies Synergistic Specification Genes That Facilitate Lineage Conversion to Prostate Tissue. Nat. Commun..

[33] Xie Q., Liu Y., Cai T., Horton C., Stefanson J., Wang Z.A. (2017). Dissecting Cell-Type-Specific Roles of Androgen Receptor in Prostate Homeostasis and Regeneration through Lineage Tracing. Nat. Commun..

[34] Dutta A., Le Magnen C., Mitrofanova A., Ouyang X., Califano A., Abate-Shen C. (2016). Identification of an NKX3.1-G9a-UTY Transcriptional Regulatory Network That Controls Prostate Differentiation. Science.

[35] Huang L., Pu Y., Hepps D., Danielpour D., Prins G.S. (2007). Posterior Hox Gene Expression and Differential Androgen Regulation in the Developing and Adult Rat Prostate Lobes. Endocrinology.

[36] Javed S., Langley S.E.M. (2014). Importance of HOX Genes in Normal Prostate Gland Formation, Prostate Cancer Development and Its Early Detection. BJU Int..

[37] Guo W., Li L., He J., Liu Z., Han M., Li F., Xia X., Zhang X., Zhu Y., Wei Y. (2020). Single-Cell Transcriptomics Identifies a Distinct Luminal Progenitor Cell Type in Distal Prostate Invagination Tips. Nat. Genet..

[38] Isaacs J.T., Rogers C.H., Coffey D.S., Cunha G., Grayhack J.T., Hinman F., Horton R. (1987). Control of cell proliferation and cell death in the normal and neoplastic prostate: A stem cell model. Benign Prostatic Hyperplasia.

[39] Tsujimura A., Koikawa Y., Salm S., Takao T., Coetzee S., Moscatelli D., Shapiro E., Lepor H., Sun T.-T., Wilson E.L. (2002). Proximal Location of Mouse Prostate Epithelial Stem Cells: A Model of Prostatic Homeostasis. J. Cell Biol..

[40] Henry G.H., Malewska A., Joseph D.B., Malladi V.S., Lee J., Torrealba J., Mauck R.J., Gahan J.C., Raj G.V., Roehrborn C.G. (2018). A Cellular Anatomy of the Normal Adult Human Prostate and Prostatic Urethra. Cell Rep..

[41] Karthaus W.R., Hofree M., Choi D., Linton E.L., Turkekul M., Bejnood A., Carver B., Gopalan A., Abida W., Laudone V. (2020). Regenerative Potential of Prostate Luminal Cells Revealed by Single-Cell Analysis. Science.

[42] Montoro D.T., Haber A.L., Biton M., Vinarsky V., Lin B., Birket S.E., Yuan F., Chen S., Leung H.M., Villoria J. (2018). A Revised Airway Epithelial Hierarchy Includes CFTR-Expressing Ionocytes. Nature.

[43] Maitland N.J., Collins A.T. (2008). Prostate Cancer Stem Cells: A New Target for Therapy. J. Clin. Oncol..

[44] Hudson D.L., Guy A.T., Fry P., O’Hare M.J., Watt F.M., Masters J.R. (2001). Epithelial Cell Differentiation Pathways in the Human Prostate: Identification of Intermediate Phenotypes by Keratin Expression. J. Histochem. Cytochem..

[45] Wang Z.A., Mitrofanova A., Bergren S.K., Abate-Shen C., Cardiff R.D., Califano A., Shen M.M. (2013). Lineage Analysis of Basal Epithelial Cells Reveals Their Unexpected Plasticity and Supports a Cell-of-Origin Model for Prostate Cancer Heterogeneity. Nat. Cell Biol..

[46] Wang X., Julio M.K., Economides K.D., Walker D., Yu H., Vivienne Halili M., Hu Y.-P., Price S.M., Abate-Shen C., Shen M.M. (2009). A Luminal Epithelial Stem Cell That Is a Cell of Origin for Prostate Cancer. Nature.

[47] Goldstein A.S., Huang J., Guo C., Garraway I.P., Witte O.N. (2010). Identification of a Cell of Origin for Human Prostate Cancer. Science.

[48] Choi N., Zhang B., Zhang L., Ittmann M., Xin L. (2012). Adult Murine Prostate Basal and Luminal Cells Are Self-Sustained Lineages That Can Both Serve as Targets for Prostate Cancer Initiation. Cancer Cell.

[49] Wang Z.A., Toivanen R., Bergren S.K., Chambon P., Shen M.M. (2014). Luminal Cells Are Favored as the Cell of Origin for Prostate Cancer. Cell Rep..

[50] Lee S.H., Shen M.M. (2015). Cell Types of Origin for Prostate Cancer. Curr. Opin. Cell Biol..

[51] Sfanos K.S., Yegnasubramanian S., Nelson W.G., De Marzo A.M. (2018). The Inflammatory Microenvironment and Microbiome in Prostate Cancer Development. Nat. Rev. Urol..

[52] Guo H., Ci X., Ahmed M., Hua J.T., Soares F., Lin D., Puca L., Vosoughi A., Xue H., Li E. (2019). ONECUT2 Is a Driver of Neuroendocrine Prostate Cancer. Nat. Commun..

[53] Giunchi F., Fiorentino M., Loda M. (2019). The Metabolic Landscape of Prostate Cancer. Eur. Urol. Oncol..

[54] Corbin J.M., Ruiz-Echevarría M.J. (2016). One-Carbon Metabolism in Prostate Cancer: The Role of Androgen Signaling. Int. J. Mol. Sci..

[55] Gurel B., Iwata T., Koh C.M., Jenkins R.B., Lan F., Van Dang C., Hicks J.L., Morgan J., Cornish T.C., Sutcliffe S. (2008). Nuclear MYC Protein Overexpression Is an Early Alteration in Human Prostate Carcinogenesis. Mod. Pathol..

[56] Civenni G., Malek A., Albino D., Garcia-Escudero R., Napoli S., Di Marco S., Pinton S., Sarti M., Carbone G.M., Catapano C.V. (2013). RNAi-Mediated Silencing of Myc Transcription Inhibits Stem-like Cell Maintenance and Tumorigenicity in Prostate Cancer. Cancer Res..

[57] Vander Griend D.J., Litvinov I.V., Isaacs J.T. (2014). Conversion of Androgen Receptor Signaling from a Growth Suppressor in Normal Prostate Epithelial Cells to an Oncogene in Prostate Cancer Cells Involves a Gain of Function in c-Myc Regulation. Int. J. Biol. Sci..

[58] Antony L., van der Schoor F., Dalrymple S.L., Isaacs J.T. (2014). Androgen Receptor (AR) Suppresses Normal Human Prostate Epithelial Cell Proliferation via AR/β-catenin/TCF-4 Complex Inhibition of c-MYC Transcription. Prostate.

[59] Maitland N.J., Collins A. (2005). A Tumour Stem Cell Hypothesis for the Origins of Prostate Cancer. BJU Int..

[60] Song H., Weinstein H.N.W., Allegakoen P., Wadsworth M.H., Xie J., Yang H., Feng F.Y., Carroll P.R., Wang B., Cooperberg M.R. (2020). Single-Cell Analysis of Human Primary Prostate Cancer Reveals the Heterogeneity of Tumor-Associated Epithelial Cell States. bioRxiv.

[61] Pomerantz M.M., Li F., Takeda D.Y., Lenci R., Chonkar A., Chabot M., Cejas P., Vazquez F., Cook J., Shivdasani R.A. (2015). The Androgen Receptor Cistrome Is Extensively Reprogrammed in Human Prostate Tumorigenesis. Nat. Genet..

[62] Grbesa I., Augello M.A., Liu D., McNally D.R., Gaffney C.D. (2021). SPOP Mutation Confers Sensitivity to AR-Targeted Therapy in Prostate Cancer by Reshaping the Androgen-Driven Chromatin Landscape. bioRxiv.

[63] Sharma N.L., Massie C.E., Ramos-Montoya A., Zecchini V., Scott H.E., Lamb A.D., MacArthur S., Stark R., Warren A.Y., Mills I.G. (2013). The Androgen Receptor Induces a Distinct Transcriptional Program in Castration-Resistant Prostate Cancer in Man. Cancer Cell.

[64] Pomerantz M.M., Qiu X., Zhu Y., Takeda D.Y., Pan W., Baca S.C., Gusev A., Korthauer K.D., Severson T.M., Ha G. (2020). Prostate Cancer Reactivates Developmental Epigenomic Programs during Metastatic Progression. Nat. Genet..

[65] Kwon O.-J., Zhang L., Jia D., Zhou Z., Li Z., Haffner M., Lee J.K., True L., Morrissey C., Xin L. (2020). De Novo Induction of Lineage Plasticity from Human Prostate Luminal Epithelial Cells by Activated AKT1 and c-Myc. Oncogene.

[66] Carceles-Cordon M., Kelly W.K., Gomella L., Knudsen K.E., Rodriguez-Bravo V., Domingo-Domenech J. (2020). Cellular Rewiring in Lethal Prostate Cancer: The Architect of Drug Resistance. Nat. Rev. Urol..

[67] Linja M.J., Savinainen K.J., Saramäki O.R., Tammela T.L., Vessella R.L., Visakorpi T. (2001). Amplification and Overexpression of Androgen Receptor Gene in Hormone-Refractory Prostate Cancer. Cancer Res..

[68] Zhang Z., Zhou C., Li X., Barnes S.D., Deng S., Hoover E., Chen C.-C., Lee Y.S., Zhang Y., Wang C. (2020). Loss of CHD1 Promotes Heterogeneous Mechanisms of Resistance to AR-Targeted Therapy via Chromatin Dysregulation. Cancer Cell.

[69] Brennen W.N., Nathaniel Brennen W., Zhu Y., Coleman I.M., Dalrymple S.L., Antony L., Patel R.A., Hanratty B., Chikarmane R., Meeker A.K. (2021). Resistance to Androgen Receptor Signaling Inhibition Does Not Necessitate Development of Neuroendocrine Prostate Cancer. JCI Insight.

[70] Nyquist M.D., Corella A., Coleman I., De Sarkar N., Kaipainen A., Ha G., Gulati R., Ang L., Chatterjee P., Lucas J. (2020). Combined TP53 and RB1 Loss Promotes Prostate Cancer Resistance to a Spectrum of Therapeutics and Confers Vulnerability to Replication Stress. Cell Rep..

[71] He M.X., Cuoco M.S., Crowdis J., Bosma-Moody A., Zhang Z., Bi K., Kanodia A., Su M.-J., Ku S.-Y., Garcia M.M. (2021). Transcriptional Mediators of Treatment Resistance in Lethal Prostate Cancer. Nat. Med..

[72] Uusi-Mäkelä J., Afyounian E., Tabaro F., Häkkinen T., Lussana A., Shcherban A., Annala M., Nurminen R., Kivinummi K., Tammela T.L.J. (2020). Chromatin Accessibility Analysis Uncovers Regulatory Element Landscape in Prostate Cancer Progression. bioRxiv.

[73] Ku S.Y., Rosario S., Wang Y., Mu P., Seshadri M., Goodrich Z.W., Goodrich M.M., Labbé D.P., Gomez E.C., Wang J. (2017). Rb1 and Trp53 Cooperate to Suppress Prostate Cancer Lineage Plasticity, Metastasis, and Antiandrogen Resistance. Science.

[74] Mandigo A.C., Yuan W., Xu K., Gallagher P., Pang A., Guan Y.F., Shafi A.A., Thangavel C., Sheehan B., Bogdan D. (2021). RB/E2F1 as a Master Regulator of Cancer Cell Metabolism in Advanced Disease. Cancer Discov..

[75] Kaarijärvi R., Kaljunen H., Ketola K. (2021). Molecular and Functional Links between Neurodevelopmental Processes and Treatment-Induced Neuroendocrine Plasticity in Prostate Cancer Progression. Cancers.

[76] Park J.W., Lee J.K., Sheu K.M., Wang L., Balanis N.G., Nguyen K., Smith B.A., Cheng C., Tsai B.L., Cheng D. (2018). Reprogramming Normal Human Epithelial Tissues to a Common, Lethal Neuroendocrine Cancer Lineage. Science.

[77] Baca S.C., Takeda D.Y., Seo J.-H., Hwang J., Ku S.Y., Arafeh R., Arnoff T., Agarwal S., Bell C., O’Connor E. (2021). Reprogramming of the FOXA1 Cistrome in Treatment-Emergent Neuroendocrine Prostate Cancer. Nat. Commun..

[78] Flores-Morales A., Bergmann T.B., Lavallee C., Batth T.S., Lin D., Lerdrup M., Friis S., Bartels A., Kristensen G., Krzyzanowska A. (2019). Proteogenomic Characterization of Patient-Derived Xenografts Highlights the Role of REST in Neuroendocrine Differentiation of Castration-Resistant Prostate Cancer. Clin. Cancer Res..

[79] Svensson C., Ceder J., Iglesias-Gato D., Chuan Y.-C., Pang S.T., Bjartell A., Martinez R.M., Bott L., Helczynski L., Ulmert D. (2014). REST Mediates Androgen Receptor Actions on Gene Repression and Predicts Early Recurrence of Prostate Cancer. Nucleic Acids Res..

[80] Prager B.C., Xie Q., Bao S., Rich J.N. (2019). Cancer Stem Cells: The Architects of the Tumor Ecosystem. Cell Stem Cell.

[81] Ge R., Wang Z., Montironi R., Jiang Z., Cheng M., Santoni M., Huang K., Massari F., Lu X., Cimadamore A. (2020). Epigenetic Modulations and Lineage Plasticity in Advanced Prostate Cancer. Ann. Oncol..

[82] Berger A., Brady N.J., Bareja R., Robinson B., Conteduca V., Augello M.A., Puca L., Ahmed A., Dardenne E., Lu X. (2019). N-Myc–mediated Epigenetic Reprogramming Drives Lineage Plasticity in Advanced Prostate Cancer. J. Clin. Investig..

[83] Chatterjee A., Rodger E.J., Eccles M.R. (2018). Epigenetic Drivers of Tumourigenesis and Cancer Metastasis. Semin. Cancer Biol..

[84] Ruggero K., Farran-Matas S., Martinez-Tebar A., Aytes A. (2018). Epigenetic Regulation in Prostate Cancer Progression. Curr. Mol. Biol. Rep..

[85] Heinäniemi M., Nykter M., Kramer R., Wienecke-Baldacchino A., Sinkkonen L., Zhou J.X., Kreisberg R., Kauffman S.A., Huang S., Shmulevich I. (2013). Gene-Pair Expression Signatures Reveal Lineage Control. Nat. Methods.

[86] Hudson T.J., Anderson W., Artez A., Barker A.D., Bell C., Bernabé R.R., Bhan M.K., Calvo F., Eerola I., International Cancer Genome Consortium (2010). International Network of Cancer Genome Projects. Nature.

[87] Robinson D., Van Allen E.M., Wu Y.-M., Schultz N., Lonigro R.J., Mosquera J.-M., Montgomery B., Taplin M.-E., Pritchard C.C., Attard G. (2015). Integrative Clinical Genomics of Advanced Prostate Cancer. Cell.

[88] Grasso C.S., Wu Y.-M., Robinson D.R., Cao X., Dhanasekaran S.M., Khan A.P., Quist M.J., Jing X., Lonigro R.J., Brenner J.C. (2012). The Mutational Landscape of Lethal Castration-Resistant Prostate Cancer. Nature.

[89] Ylipää A., Kivinummi K., Kohvakka A., Annala M., Latonen L., Scaravilli M., Kartasalo K., Leppänen S.-P., Karakurt S., Seppälä J. (2015). Transcriptome Sequencing Reveals PCAT5 as a Novel ERG-Regulated Long Noncoding RNA in Prostate Cancer. Cancer Res..

[90] Han Y.-C., Zheng Z.-L., Zuo Z.-H., Yu Y.P., Chen R., Tseng G.C., Nelson J.B., Luo J.-H. (2013). Metallothionein 1 H Tumour Suppressor Activity in Prostate Cancer Is Mediated by Euchromatin Methyltransferase 1. J. Pathol..

[91] Augello M.A., Hickey T.E., Knudsen K.E. (2011). FOXA1: Master of Steroid Receptor Function in Cancer. EMBO J..

[92] Teng M., Zhou S., Cai C., Lupien M., He H.H. (2021). Pioneer of Prostate Cancer: Past, Present and the Future of FOXA1. Protein Cell.

[93] Lupien M., Eeckhoute J., Meyer C.A., Wang Q., Zhang Y., Li W., Carroll J.S., Liu X.S., Brown M. (2008). FoxA1 Translates Epigenetic Signatures into Enhancer-Driven Lineage-Specific Transcription. Cell.

[94] Heinlein C.A., Chang C. (2004). Androgen Receptor in Prostate Cancer. Endocr. Rev..

[95] Rao R.C., Dou Y. (2015). Hijacked in Cancer: The KMT2 (MLL) Family of Methyltransferases. Nat. Rev. Cancer.

[96] Stokes D.G., Tartof K.D., Perry R.P. (1996). CHD1 Is Concentrated in Interbands and Puffed Regions of Drosophila Polytene Chromosomes. Proc. Natl. Acad. Sci. USA.

[97] Boysen G., Rodrigues D.N., Rescigno P., Seed G., Dolling D., Riisnaes R., Crespo M., Zafeiriou Z., Sumanasuriya S., Bianchini D. (2018). SPOP-Mutated/CHD1-Deleted Lethal Prostate Cancer and Abiraterone Sensitivity. Clin. Cancer Res..

[98] Shenoy T.R., Boysen G., Wang M.Y., Xu Q.Z., Guo W., Koh F.M., Wang C., Zhang L.Z., Wang Y., Gil V. (2017). CHD1 Loss Sensitizes Prostate Cancer to DNA Damaging Therapy by Promoting Error-Prone Double-Strand Break Repair. Ann. Oncol..

[99] Aparicio A.M., Shen L., Tapia E.L.N., Lu J.-F., Chen H.-C., Zhang J., Wu G., Wang X., Troncoso P., Corn P. (2016). Combined Tumor Suppressor Defects Characterize Clinically Defined Aggressive Variant Prostate Cancers. Clin. Cancer Res..

[100] Mazrooei P., Kron K.J., Zhu Y., Zhou S., Grillo G., Mehdi T., Ahmed M., Severson T.M., Guilhamon P., Armstrong N.S. (2019). Cistrome Partitioning Reveals Convergence of Somatic Mutations and Risk Variants on Master Transcription Regulators in Primary Prostate Tumors. Cancer Cell.

[101] Takeda D.Y., Spisák S., Seo J.-H., Bell C., O’Connor E., Korthauer K., Ribli D., Csabai I., Solymosi N., Szállási Z. (2018). A Somatically Acquired Enhancer of the Androgen Receptor Is a Noncoding Driver in Advanced Prostate Cancer. Cell.

[102] Lee W.H., Morton R.A., Epstein J.I., Brooks J.D., Campbell P.A., Bova G.S., Hsieh W.S., Isaacs W.B., Nelson W.G. (1994). Cytidine Methylation of Regulatory Sequences near the Pi-Class Glutathione S-Transferase Gene Accompanies Human Prostatic Carcinogenesis. Proc. Natl. Acad. Sci. USA.

[103] Lee W.H., Isaacs W.B., Bova G.S., Nelson W.G. (1997). CG Island Methylation Changes near the GSTP1 Gene in Prostatic Carcinoma Cells Detected Using the Polymerase Chain Reaction: A New Prostate Cancer Biomarker. Cancer Epidemiol. Biomark. Prev..

[104] Martignano F., Gurioli G., Salvi S., Calistri D., Costantini M., Gunelli R., De Giorgi U., Foca F., Casadio V. (2016). GSTP1 Methylation and Protein Expression in Prostate Cancer: Diagnostic Implications. Dis. Markers.

[105] Mahapatra S., Klee E.W., Young C.Y.F., Sun Z., Jimenez R.E., Klee G.G., Tindall D.J., Donkena K.V. (2012). Global Methylation Profiling for Risk Prediction of Prostate Cancer. Clin. Cancer Res..

[106] Börno S.T., Fischer A., Kerick M., Fälth M., Laible M. (2012). Genome-Wide DNA Methylation Events in TMPRSS2–ERG Fusion-Negative Prostate Cancers Implicate an EZH2-Dependent Mechanism with miR-26a Hypermethylation. Cancer Discov..

[107] Friedlander T.W., Roy R., Tomlins S.A., Ngo V.T., Kobayashi Y., Azameera A., Rubin M.A., Pienta K.J., Chinnaiyan A., Ittmann M.M. (2012). Common Structural and Epigenetic Changes in the Genome of Castration-Resistant Prostate Cancer. Cancer Res..

[108] Zhao S.G., Chen W.S., Li H., Foye A., Zhang M., Sjöström M., Aggarwal R., Playdle D., Liao A., Alumkal J.J. (2020). The DNA Methylation Landscape of Advanced Prostate Cancer. Nat. Genet..

[109] Kim J.H., Dhanasekaran S.M., Prensner J.R., Cao X., Robinson D., Kalyana-Sundaram S., Huang C., Shankar S., Jing X., Iyer M. (2011). Deep Sequencing Reveals Distinct Patterns of DNA Methylation in Prostate Cancer. Genome Res..

[110] Kron K., Pethe V., Briollais L., Sadikovic B., Ozcelik H., Sunderji A., Venkateswaran V., Pinthus J., Fleshner N., van der Kwast T. (2009). Discovery of Novel Hypermethylated Genes in Prostate Cancer Using Genomic CpG Island Microarrays. PLoS ONE.

[111] Kron K., Trudel D., Pethe V., Briollais L., Fleshner N., van der Kwast T., Bapat B. (2013). Altered DNA Methylation Landscapes of Polycomb-Repressed Loci Are Associated with Prostate Cancer Progression and ERG Oncogene Expression in Prostate Cancer. Clin. Cancer Res..

[112] Jones P.A., Baylin S.B. (2002). The Fundamental Role of Epigenetic Events in Cancer. Nat. Rev. Genet..

[113] Smith Z.D., Meissner A. (2013). DNA Methylation: Roles in Mammalian Development. Nat. Rev. Genet..

[114] Parry A., Rulands S., Reik W. (2021). Active Turnover of DNA Methylation during Cell Fate Decisions. Nat. Rev. Genet..

[115] Greenberg M.V.C., Bourc’his D. (2019). The Diverse Roles of DNA Methylation in Mammalian Development and Disease. Nat. Rev. Mol. Cell Biol..

[116] Massie C.E., Mills I.G., Lynch A.G. (2017). The Importance of DNA Methylation in Prostate Cancer Development. J. Steroid Biochem. Mol. Biol..

[117] Alvarez S., Germain P., Alvarez R., Rodríguez-Barrios F., Gronemeyer H., de Lera A.R. (2007). Structure, Function and Modulation of Retinoic Acid Receptor Beta, a Tumor Suppressor. Int. J. Biochem. Cell Biol..

[118] Wang W., Liu S., Jiang C., Wang Y., Zhu H., Wang X. (2019). High Expression of RARβ Is a Favorable Factor in Colorectal Cancer. Dis. Markers.

[119] Buijs J.T., Rentsch C.A., van der Horst G., van Overveld P.G.M., Wetterwald A., Schwaninger R., Henriquez N.V., Ten Dijke P., Borovecki F., Markwalder R. (2007). BMP7, a Putative Regulator of Epithelial Homeostasis in the Human Prostate, Is a Potent Inhibitor of Prostate Cancer Bone Metastasis in Vivo. Am. J. Pathol..

[120] Panja S., Hayati S., Epsi N.J., Parrott J.S., Mitrofanova A. (2018). Integrative (epi) genomic analysis to predict response to androgen-deprivation therapy in prostate cancer. EBioMedicine.

[121] Fiano V., Zugna D., Grasso C., Trevisan M., Delsedime L., Molinaro L., Gillio-Tos A., Merletti F., Richiardi L. (2017). LINE-1 Methylation Status in Prostate Cancer and Non-Neoplastic Tissue Adjacent to Tumor in Association with Mortality. Epigenetics.

[122] Storebjerg T.M., Strand S.H., Høyer S., Lynnerup A.-S., Borre M., Ørntoft T.F., Sørensen K.D. (2018). Dysregulation and Prognostic Potential of 5-Methylcytosine (5mC), 5-Hydroxymethylcytosine (5hmC), 5-Formylcytosine (5fC), and 5-Carboxylcytosine (5caC) Levels in Prostate Cancer. Clin. Epigenet..

[123] The Cancer Genome Atlas Research Network (2015). The Molecular Taxonomy of Primary Prostate Cancer. Cell.

[124] Noushmehr H., Weisenberger D.J., Diefes K., Phillips H.S., Pujara K., Berman B.P., Pan F., Pelloski C.E., Sulman E.P., Bhat K.P. (2010). Identification of a CpG Island Methylator Phenotype That Defines a Distinct Subgroup of Glioma. Cancer Cell.

[125] Gerhauser C., Favero F., Risch T., Simon R., Feuerbach L., Assenov Y., Heckmann D., Sidiropoulos N., Waszak S.M., Hübschmann D. (2018). Molecular Evolution of Early-Onset Prostate Cancer Identifies Molecular Risk Markers and Clinical Trajectories. Cancer Cell.

[126] Brunskill E.W., Sequeira-Lopez M.L.S., Pentz E.S., Lin E., Yu J., Aronow B.J., Potter S.S., Gomez R.A. (2011). Genes That Confer the Identity of the Renin Cell. J. Am. Soc. Nephrol..

[127] Ebihara T., Song C., Ryu S.H., Plougastel-Douglas B., Yang L., Levanon D., Groner Y., Bern M.D., Stappenbeck T.S., Colonna M. (2015). Runx3 Specifies Lineage Commitment of Innate Lymphoid Cells. Nat. Immunol..

[128] Ruiz S., Santos E., Bustelo X.R. (2007). RasGRF2, a Guanosine Nucleotide Exchange Factor for Ras GTPases, Participates in T-Cell Signaling Responses. Mol. Cell. Biol..

[129] Wang Z., Deng T., Long X., Lin X., Wu S., Wang H., Ge R., Zhang Z., Wu C.-L., Taplin M.-E. (2020). Methylation of SRD5A2 Promoter Predicts a Better Outcome for Castration-Resistant Prostate Cancer Patients Undergoing Androgen Deprivation Therapy. PLoS ONE.

[130] Peter M.R., Bilenky M., Davies A., Isserlin R., Bader G.D., Fleshner N.E., Hirst M., Zoubeidi A., Bapat B. (2021). Distinct DNA Methylation Patterns Associated with Treatment Resistance in Metastatic Castration Resistant Prostate Cancer. Sci. Rep..

[131] Hyun K., Jeon J., Park K., Kim J. (2017). Writing, Erasing and Reading Histone Lysine Methylations. Exp. Mol. Med..

[132] Bannister A.J., Kouzarides T. (2011). Regulation of chromatin by histone modifications. Cell Res..

[133] Ernst J., Kellis M. (2012). ChromHMM: Automating Chromatin-State Discovery and Characterization. Nat. Methods.

[134] Ernst J., Kellis M. (2017). Chromatin-State Discovery and Genome Annotation with ChromHMM. Nat. Protoc..

[135] Kundaje A., Meuleman W., Ernst J., Bilenky M., Yen A., Heravi-Moussavi A., Kheradpour P., Zhang Z., Wang J., Roadmap Epigenomics Consortium (2015). Integrative Analysis of 111 Reference Human Epigenomes. Nature.

[136] Chen Z., Wang L., Wang Q., Li W. (2010). Histone Modifications and Chromatin Organization in Prostate Cancer. Epigenomics.

[137] Kang Z., Jänne O.A., Palvimo J.J. (2004). Coregulator Recruitment and Histone Modifications in Transcriptional Regulation by the Androgen Receptor. Mol. Endocrinol..

[138] Wang Q., Carroll J.S., Brown M. (2005). Spatial and Temporal Recruitment of Androgen Receptor and Its Coactivators Involves Chromosomal Looping and Polymerase Tracking. Mol. Cell.

[139] Shang Y., Myers M., Brown M. (2002). Formation of the Androgen Receptor Transcription Complex. Mol. Cell.

[140] Zhong J., Ding L., Bohrer L.R., Pan Y., Liu P., Zhang J., Sebo T.J., Karnes R.J., Tindall D.J., van Deursen J. (2014). p300 Acetyltransferase Regulates Androgen Receptor Degradation and PTEN-Deficient Prostate Tumorigenesis. Cancer Res..

[141] Liu J., He D., Cheng L., Huang C., Zhang Y., Rao X., Kong Y., Li C., Zhang Z., Liu J. (2020). p300/CBP Inhibition Enhances the Efficacy of Programmed Death-Ligand 1 Blockade Treatment in Prostate Cancer. Oncogene.

[142] Yamane K., Toumazou C., Tsukada Y.-I., Erdjument-Bromage H., Tempst P., Wong J., Zhang Y. (2006). JHDM2A, a JmjC-Containing H3K9 Demethylase, Facilitates Transcription Activation by Androgen Receptor. Cell.

[143] Wissmann M., Yin N., Müller J.M., Greschik H., Fodor B.D., Jenuwein T., Vogler C., Schneider R., Günther T., Buettner R. (2007). Cooperative Demethylation by JMJD2C and LSD1 Promotes Androgen Receptor-Dependent Gene Expression. Nat. Cell Biol..

[144] Metzger E., Wissmann M., Yin N., Müller J.M., Schneider R., Peters A.H.F.M., Günther T., Buettner R., Schüle R. (2005). LSD1 Demethylates Repressive Histone Marks to Promote Androgen-Receptor-Dependent Transcription. Nature.

[145] Seligson D.B., Horvath S., McBrian M.A., Mah V., Yu H., Tze S., Wang Q., Chia D., Goodglick L., Kurdistani S.K. (2009). Global Levels of Histone Modifications Predict Prognosis in Different Cancers. Am. J. Pathol..

[146] Ellinger J., Kahl P., von der Gathen J., Rogenhofer S., Heukamp L.C., Gütgemann I., Walter B., Hofstädter F., Büttner R., Müller S.C. (2010). Global Levels of Histone Modifications Predict Prostate Cancer Recurrence. Prostate.

[147] Bianco-Miotto T., Chiam K., Buchanan G., Jindal S., Day T.K., Thomas M., Pickering M.A., O’Loughlin M.A., Ryan N.K., Raymond W.A. (2010). Global Levels of Specific Histone Modifications and an Epigenetic Gene Signature Predict Prostate Cancer Progression and Development. Cancer Epidemiol. Biomark. Prev..

[148] Ke X.-S., Qu Y., Rostad K., Li W.-C., Lin B., Halvorsen O.J., Haukaas S.A., Jonassen I., Petersen K., Goldfinger N. (2009). Genome-Wide Profiling of Histone h3 Lysine 4 and Lysine 27 Trimethylation Reveals an Epigenetic Signature in Prostate Carcinogenesis. PLoS ONE.

[149] Gal-Yam E.N., Egger G., Iniguez L., Holster H., Einarsson S., Zhang X., Lin J.C., Liang G., Jones P.A., Tanay A. (2008). Frequent Switching of Polycomb Repressive Marks and DNA Hypermethylation in the PC3 Prostate Cancer Cell Line. Proc. Natl. Acad. Sci. USA.

[150] Welti J., Sharp A., Brooks N., Yuan W., McNair C., Chand S.N., Pal A., Figueiredo I., Riisnaes R., Gurel B. (2021). Targeting the p300/CBP Axis in Lethal Prostate Cancer. Cancer Discov..

[151] Xu S., Fan L., Jeon H.-Y., Zhang F., Cui X., Mickle M.B., Peng G., Hussain A., Fazli L., Gleave M.E. (2020). p300-Mediated Acetylation of Histone Demethylase JMJD1A Prevents Its Degradation by Ubiquitin Ligase STUB1 and Enhances Its Activity in Prostate Cancer. Cancer Res..

[152] Sahu B., Laakso M., Ovaska K., Mirtti T., Lundin J., Rannikko A., Sankila A., Turunen J.-P., Lundin M., Konsti J. (2011). Dual Role of FoxA1 in Androgen Receptor Binding to Chromatin, Androgen Signalling and Prostate Cancer. EMBO J..

[153] Wang Q., Li W., Zhang Y., Yuan X., Xu K., Yu J., Chen Z., Beroukhim R., Wang H., Lupien M. (2009). Androgen Receptor Regulates a Distinct Transcription Program in Androgen-Independent Prostate Cancer. Cell.

[154] Pellakuru L.G., Iwata T., Gurel B., Schultz D., Hicks J., Bethel C., Yegnasubramanian S., De Marzo A.M. (2012). Global Levels of H3K27me3 Track with Differentiation in Vivo and Are Deregulated by MYC in Prostate Cancer. Am. J. Pathol..

[155] Yu J., Cao Q., Mehra R., Laxman B., Yu J., Tomlins S.A., Creighton C.J., Dhanasekaran S.M., Shen R., Chen G. (2007). Integrative Genomics Analysis Reveals Silencing of Beta-Adrenergic Signaling by Polycomb in Prostate Cancer. Cancer Cell.

[156] Yu J., Yu J., Rhodes D.R., Tomlins S.A., Cao X., Chen G., Mehra R., Wang X., Ghosh D., Shah R.B. (2007). A Polycomb Repression Signature in Metastatic Prostate Cancer Predicts Cancer Outcome. Cancer Res..

[157] Bryant R.J., Cross N.A., Eaton C.L., Hamdy F.C., Cunliffe V.T. (2007). EZH2 Promotes Proliferation and Invasiveness of Prostate Cancer Cells. Prostate.

[158] Dundr P., Bártů M., Hojný J., Michálková R., Hájková N., Stružinská I., Krkavcová E., Hadravský L., Kleissnerová L., Kopejsková J. (2020). HNF1B, EZH2 and ECI2 in Prostate Carcinoma. Molecular, Immunohistochemical and Clinico-Pathological Study. Sci. Rep..

[159] Varambally S., Dhanasekaran S.M., Zhou M., Barrette T.R., Kumar-Sinha C., Sanda M.G., Ghosh D., Pienta K.J., Sewalt R.G.A.B., Otte A.P. (2002). The Polycomb Group Protein EZH2 Is Involved in Progression of Prostate Cancer. Nature.

[160] Melling N., Thomsen E., Tsourlakis M.C., Kluth M., Hube-Magg C., Minner S., Koop C., Graefen M., Heinzer H., Wittmer C. (2015). Overexpression of Enhancer of Zeste Homolog 2 (EZH2) Characterizes an Aggressive Subset of Prostate Cancers and Predicts Patient Prognosis Independently from Pre- and Postoperatively Assessed Clinicopathological Parameters. Carcinogenesis.

[161] Xu K., Wu Z.J., Groner A.C., He H.H., Cai C., Lis R.T., Wu X., Stack E.C., Loda M., Liu T. (2012). EZH2 Oncogenic Activity in Castration-Resistant Prostate Cancer Cells Is Polycomb-Independent. Science.

[162] Clapier C.R., Iwasa J., Cairns B.R., Peterson C.L. (2017). Mechanisms of Action and Regulation of ATP-Dependent Chromatin-Remodelling Complexes. Nat. Rev. Mol. Cell Biol..

[163] Hargreaves D.C. (2021). Chromatin Openness Requires Continuous SWI/SNF Activity. Nat. Genet..

[164] Cyrta J., Augspach A., De Filippo M.R., Prandi D., Thienger P., Benelli M., Cooley V., Bareja R., Wilkes D., Chae S.-S. (2020). Role of Specialized Composition of SWI/SNF Complexes in Prostate Cancer Lineage Plasticity. Nat. Commun..

[165] Giles K.A., Gould C.M., Achinger-Kawecka J., Page S.G., Kafer G.R., Rogers S., Luu P.-L., Cesare A.J., Clark S.J., Taberlay P.C. (2021). BRG1 Knockdown Inhibits Proliferation through Multiple Cellular Pathways in Prostate Cancer. Clin. Epigenet..

[166] Sun A., Tawfik O., Gayed B., Thrasher J.B., Hoestje S., Li C., Li B. (2007). Aberrant Expression of SWI/SNF Catalytic Subunits BRG1/BRM Is Associated with Tumor Development and Increased Invasiveness in Prostate Cancers. Prostate.

[167] Ding Y., Li N., Dong B., Guo W., Wei H., Chen Q., Yuan H., Han Y., Chang H., Kan S. (2019). Chromatin Remodeling ATPase BRG1 and PTEN Are Synthetic Lethal in Prostate Cancer. J. Clin. Investig..

[168] Muthuswami R., Bailey L., Rakesh R., Imbalzano A.N., Nickerson J.A., Hockensmith J.W. (2019). BRG1 Is a Prognostic Indicator and a Potential Therapeutic Target for Prostate Cancer. J. Cell. Physiol..

[169] Jamaspishvili T., Berman D.M., Ross A.E., Scher H.I., De Marzo A.M., Squire J.A., Lotan T.L. (2018). Clinical Implications of PTEN Loss in Prostate Cancer. Nat. Rev. Urol..

[170] Balasubramaniam S., Comstock C.E.S., Ertel A., Jeong K.W., Stallcup M.R., Addya S., McCue P.A., Ostrander W.F., Augello M.A., Knudsen K.E. (2013). Aberrant BAF57 Signaling Facilitates Prometastatic Phenotypes. Clin. Cancer Res..

[171] Heebøll S., Borre M., Ottosen P.D., Andersen C.L., Mansilla F., Dyrskjøt L., Orntoft T.F., Tørring N. (2008). SMARCC1 Expression Is Upregulated in Prostate Cancer and Positively Correlated with Tumour Recurrence and Dedifferentiation. Histol. Histopathol..

[172] Link K.A., Balasubramaniam S., Sharma A., Comstock C.E.S., Godoy-Tundidor S., Powers N., Cao K.H., Haelens A., Claessens F., Revelo M.P. (2008). Targeting the BAF57 SWI/SNF Subunit in Prostate Cancer: A Novel Platform to Control Androgen Receptor Activity. Cancer Res..

[173] Stelloo S., Nevedomskaya E., van der Poel H.G., de Jong J., van Leenders G.J.L.H., Jenster G., Wessels L.F.A., Bergman A.M., Zwart W. (2015). Androgen Receptor Profiling Predicts Prostate Cancer Outcome. EMBO Mol. Med..

[174] Xu Q., Liu X., Zhu S., Hu X., Niu H., Zhang X., Zhu D., Nesa E.U., Tian K., Yuan H. (2018). Hyper-Acetylation Contributes to the Sensitivity of Chemo-Resistant Prostate Cancer Cells to Histone Deacetylase Inhibitor Trichostatin A. J. Cell. Mol. Med..

[175] Seligson D.B., Horvath S., Shi T., Yu H., Tze S., Grunstein M., Kurdistani S.K. (2005). Global Histone Modification Patterns Predict Risk of Prostate Cancer Recurrence. Nature.

[176] Zhou L.-X., Li T., Huang Y.-R., Sha J.-J., Sun P., Li D. (2010). Application of Histone Modification in the Risk Prediction of the Biochemical Recurrence after Radical Prostatectomy. Asian J. Androl..

[177] Devaiah B.N., Case-Borden C., Gegonne A., Hsu C.H., Chen Q., Meerzaman D., Dey A., Ozato K., Singer D.S. (2016). BRD4 Is a Histone Acetyltransferase That Evicts Nucleosomes from Chromatin. Nat. Struct. Mol. Biol..

[178] Surface L.E., Fields P.A., Subramanian V., Behmer R., Udeshi N., Peach S.E., Carr S.A., Jaffe J.D., Boyer L.A. (2016). H2A.Z.1 Monoubiquitylation Antagonizes BRD2 to Maintain Poised Chromatin in ESCs. Cell Rep..

[179] Stathis A., Bertoni F. (2018). BET Proteins as Targets for Anticancer Treatment. Cancer Discov..

[180] Asangani I.A., Dommeti V.L., Wang X., Malik R., Cieslik M., Yang R., Escara-Wilke J., Wilder-Romans K., Dhanireddy S., Engelke C. (2014). Therapeutic Targeting of BET Bromodomain Proteins in Castration-Resistant Prostate Cancer. Nature.

[181] Asangani I.A., Wilder-Romans K., Dommeti V.L., Krishnamurthy P.M., Apel I.J., Escara-Wilke J., Plymate S.R., Navone N.M., Wang S., Feng F.Y. (2016). BET Bromodomain Inhibitors Enhance Efficacy and Disrupt Resistance to AR Antagonists in the Treatment of Prostate Cancer. Mol. Cancer Res..

[182] Raina K., Lu J., Qian Y., Altieri M., Gordon D., Rossi A.M.K., Wang J., Chen X., Dong H., Siu K. (2016). PROTAC-Induced BET Protein Degradation as a Therapy for Castration-Resistant Prostate Cancer. Proc. Natl. Acad. Sci. USA.

[183] Wyce A., Degenhardt Y., Bai Y., Le B.C., Korenchuk S. (2013). Inhibition of BET Bromodomain Proteins as a Therapeutic Approach in Prostate Cancer. Oncotarget.

[184] Blee A.M., Liu S., Wang L., Huang H. (2016). BET Bromodomain-Mediated Interaction between ERG and BRD4 Promotes Prostate Cancer Cell Invasion. Oncotarget.

[185] Shafran J.S., Andrieu G.P., Györffy B., Denis G.V. (2019). BRD4 Regulates Metastatic Potential of Castration-Resistant Prostate Cancer through AHNAK. Mol. Cancer Res..

[186] Faivre E.J., McDaniel K.F., Albert D.H., Mantena S.R. (2020). Selective Inhibition of the BD2 Bromodomain of BET Proteins in Prostate Cancer. Nature.

[187] Huang Z.-Q., Li J., Sachs L.M., Cole P.A., Wong J. (2003). A Role for Cofactor-Cofactor and Cofactor-Histone Interactions in Targeting p300, SWI/SNF and Mediator for Transcription. EMBO J..

[188] Jin M.L., Kim Y.W., Jeong K.W. (2018). BAF53A Regulates Androgen Receptor-Mediated Gene Expression and Proliferation in LNCaP Cells. Biochem. Biophys. Res. Commun..

[189] Link K.A., Burd C.J., Williams E., Marshall T., Rosson G., Henry E., Weissman B., Knudsen K.E. (2005). BAF57 Governs Androgen Receptor Action and Androgen-Dependent Proliferation through SWI/SNF. Mol. Cell. Biol..

[190] Marshall T.W., Link K.A., Petre-Draviam C.E., Knudsen K.E. (2003). Differential Requirement of SWI/SNF for Androgen Receptor Activity. J. Biol. Chem..

[191] Urbanucci A., Mills I.G. (2018). Bromodomain-Containing Proteins in Prostate Cancer. Mol. Cell. Endocrinol..

[192] Klemm S.L., Shipony Z., Greenleaf W.J. (2019). Chromatin accessibility and the regulatory epigenome. Nat. Rev. Genet..

[193] Corces M.R., Granja J.M., Shams S., Louie B.H., Seoane J.A., Zhou W., Silva T.C., Groeneveld C., Wong C.K., Cho S.W. (2018). The Chromatin Accessibility Landscape of Primary Human Cancers. Science.

[194] Gasperini M., Tome J.M., Shendure J. (2020). Towards a Comprehensive Catalogue of Validated and Target-Linked Human Enhancers. Nat. Rev. Genet..

[195] Baek S., Goldstein I., Hager G.L. (2017). Bivariate Genomic Footprinting Detects Changes in Transcription Factor Activity. Cell Rep..

[196] Mills I.G. (2014). Maintaining and Reprogramming Genomic Androgen Receptor Activity in Prostate Cancer. Nat. Rev. Cancer.

[197] Braadland P.R., Urbanucci A. (2019). Chromatin Reprogramming as an Adaptation Mechanism in Advanced Prostate Cancer. Endocr. Relat. Cancer.

[198] Urbanucci A., Sahu B., Seppälä J., Larjo A., Latonen L.M., Waltering K.K., Tammela T.L.J., Vessella R.L., Lähdesmäki H., Jänne O.A. (2012). Overexpression of Androgen Receptor Enhances the Binding of the Receptor to the Chromatin in Prostate Cancer. Oncogene.

[199] Senapati D., Kumari S., Heemers H.V. (2020). Androgen Receptor Co-Regulation in Prostate Cancer. Asian J. Urol..

[200] Tomlins S.A., Rhodes D.R., Perner S., Dhanasekaran S.M., Mehra R., Sun X.-W., Varambally S., Cao X., Tchinda J., Kuefer R. (2005). Recurrent Fusion of TMPRSS2 and ETS Transcription Factor Genes in Prostate Cancer. Science.

[201] Yu J., Yu J., Mani R.-S., Cao Q., Brenner C.J., Cao X., Wang X., Wu L., Li J., Hu M. (2010). An Integrated Network of Androgen Receptor, Polycomb, and TMPRSS2-ERG Gene Fusions in Prostate Cancer Progression. Cancer Cell.

[202] Chng K.R., Chang C.W., Tan S.K., Yang C., Hong S.Z., Sng N.Y.W., Cheung E. (2012). A Transcriptional Repressor Co-Regulatory Network Governing Androgen Response in Prostate Cancers: Corepressor Regulation of AR Signalling. EMBO J..

[203] Chen Y., Chi P., Rockowitz S., Iaquinta P.J., Shamu T., Shukla S., Gao D., Sirota I., Carver B.S., Wongvipat J. (2013). ETS Factors Reprogram the Androgen Receptor Cistrome and Prime Prostate Tumorigenesis in Response to PTEN Loss. Nat. Med..

[204] Kron K.J., Murison A., Zhou S., Huang V., Yamaguchi T.N., Shiah Y.-J., Fraser M., van der Kwast T., Boutros P.C., Bristow R.G. (2017). TMPRSS2--ERG Fusion Co-Opts Master Transcription Factors and Activates NOTCH Signaling in Primary Prostate Cancer. Nat. Genet..

[205] Li F., Yuan Q., Di W., Xia X., Liu Z., Mao N., Li L., Li C., He J., Li Y. (2020). ERG Orchestrates Chromatin Interactions to Drive Prostate Cell Fate Reprogramming. J. Clin. Investig..

[206] Weischenfeldt J., Simon R., Feuerbach L., Schlangen K., Weichenhan D., Minner S., Wuttig D., Warnatz H.-J., Stehr H., Rausch T. (2013). Integrative Genomic Analyses Reveal an Androgen-Driven Somatic Alteration Landscape in Early-Onset Prostate Cancer. Cancer Cell.

[207] Koh C.M., Bieberich C.J., Dang C.V., Nelson W.G., Yegnasubramanian S., De Marzo A.M. (2010). MYC and Prostate Cancer. Genes Cancer.

[208] Barfeld S.J., Urbanucci A., Itkonen H.M., Fazli L., Hicks J.L., Thiede B., Rennie P.S., Yegnasubramanian S., DeMarzo A.M., Mills I.G. (2017). C-Myc Antagonises the Transcriptional Activity of the Androgen Receptor in Prostate Cancer Affecting Key Gene Networks. EBioMedicine.

[209] Qiu X., Boufaied N., Hallal T., Feit A., de Polo A., Luoma A.M., Larocque J., Zadra G., Xie Y., Gu S. (2021). MYC Drives Aggressive Prostate Cancer by Disrupting Transcriptional Pause Release at Androgen Receptor Targets. bioRxiv.

[210] He Y., Wei T., Ye Z., Orme J.J., Lin D., Sheng H., Fazli L., Karnes R.J., Jimenez R., Wang L. (2021). A Noncanonical AR Addiction Drives Enzalutamide Resistance in Prostate Cancer. Nat. Commun..

[211] Taavitsainen S., Engedal N., Cao S., Handle F., Prekovic S., Wetterskog D., Vuorinen E.M., Kiviaho A., Nätkin R., Devlies W. (2021). Single-Cell ATAC and RNA Sequencing Reveal Pre-Existing and Persistent Subpopulations of Cells Associated with Relapse of Prostate Cancer. bioRxiv.

[212] Ayaz G., Razizadeh N., Yaşar P., Kars G., Kahraman D.C., Saatci Ö., Şahin Ö., Çetin-Atalay R., Muyan M. (2020). CXXC5 as an Unmethylated CpG Dinucleotide Binding Protein Contributes to Estrogen-Mediated Cellular Proliferation. Sci. Rep..

[213] Solary E., Bernard O.A., Tefferi A., Fuks F., Vainchenker W. (2014). The Ten-Eleven Translocation-2 (TET2) Gene in Hematopoiesis and Hematopoietic Diseases. Leukemia.

[214] McAuley E., Moline D., VanOpstall C., Lamperis S., Brown R., Vander Griend D.J. (2019). Sox2 Expression Marks Castration-Resistant Progenitor Cells in the Adult Murine Prostate. Stem Cells.

[215] Mevel R., Steiner I., Mason S., Galbraith L.C.A., Patel R., Fadlullah M.Z.H., Ahmad I., Leung H.Y., Oliveira P., Blyth K. (2020). RUNX1 Marks a Luminal Castration-Resistant Lineage Established at the Onset of Prostate Development. eLife.

[216] Shah N., Wang P., Wongvipat J., Karthaus W.R., Abida W., Armenia J., Rockowitz S., Drier Y., Bernstein B.E., Long H.W. (2017). Regulation of the Glucocorticoid Receptor via a BET-Dependent Enhancer Drives Antiandrogen Resistance in Prostate Cancer. eLife.

[217] Hepburn A.C., Steele R.E., Veeratterapillay R., Wilson L., Kounatidou E.E., Barnard A., Berry P., Cassidy J.R., Moad M., El-Sherif A. (2019). The Induction of Core Pluripotency Master Regulators in Cancers Defines Poor Clinical Outcomes and Treatment Resistance. Oncogene.

[218] Dixon J.R., Gorkin D.U., Ren B. (2016). Chromatin Domains: The Unit of Chromosome Organization. Mol. Cell.

[219] Misteli T., Finn E.H. (2021). Chromatin Architecture Is a Flexible Foundation for Gene Expression. Nat. Genet..

[220] Achinger-Kawecka J., Taberlay P.C., Clark S.J. (2016). Alterations in Three-Dimensional Organization of the Cancer Genome and Epigenome. Cold Spring Harb. Symp. Quant. Biol..

[221] Taberlay P.C., Achinger-Kawecka J., Lun A.T.L., Buske F.A., Sabir K., Gould C.M., Zotenko E., Bert S.A., Giles K.A., Bauer D.C. (2016). Three-Dimensional Disorganization of the Cancer Genome Occurs Coincident with Long-Range Genetic and Epigenetic Alterations. Genome Res..

[222] Rhie S.K., Perez A.A., Lay F.D., Schreiner S., Shi J., Polin J., Farnham P.J. (2019). A High-Resolution 3D Epigenomic Map Reveals Insights into the Creation of the Prostate Cancer Transcriptome. Nat. Commun..

[223] Taslim C., Chen Z., Huang K., Huang T.H.-M., Wang Q., Lin S. (2012). Integrated Analysis Identifies a Class of Androgen-Responsive Genes Regulated by Short Combinatorial Long-Range Mechanism Facilitated by CTCF. Nucleic Acids Res..

[224] Chen Z., Zhang C., Wu D., Chen H., Rorick A., Zhang X., Wang Q. (2011). Phospho-MED1-Enhanced UBE2C Locus Looping Drives Castration-Resistant Prostate Cancer Growth: MED1 Phosphorylation Enhances DNA Looping. EMBO J..

[225] Lee C.H., Ku J.Y., Ha J.M., Bae S.S., Lee J.Z., Kim C.-S., Ha H.K. (2017). Transcript Levels of Androgen Receptor Variant 7 and Ubiquitin-Conjugating Enzyme 2C in Hormone Sensitive Prostate Cancer and Castration-Resistant Prostate Cancer. Prostate.

[226] Wang Y., Wang J., Tang Q., Ren G. (2021). Identification of UBE2C as Hub Gene in Driving Prostate Cancer by Integrated Bioinformatics Analysis. PLoS ONE.

[227] Hu Y., Gu Y., Wang H., Huang Y., Zou Y.M. (2015). Integrated Network Model Provides New Insights into Castration-Resistant Prostate Cancer. Sci. Rep..

[228] Wang H., Zhang C., Rorick A., Wu D., Chiu M. (2011). CCI-779 Inhibits Cell-Cycle G2–M Progression and Invasion of Castration-Resistant Prostate Cancer via Attenuation of UBE2C Transcription and mRNA Stability. Cancer Res..

[229] Liu G., Sprenger C., Wu P.-J., Sun S., Uo T., Haugk K., Epilepsia K.S., Plymate S. (2015). MED1 Mediates Androgen Receptor Splice Variant Induced Gene Expression in the Absence of Ligand. Oncotarget.

[230] Du M., Tillmans L., Gao J., Gao P., Yuan T., Dittmar R.L., Song W., Yang Y., Sahr N., Wang T. (2016). Chromatin Interactions and Candidate Genes at Ten Prostate Cancer Risk Loci. Sci. Rep..

[231] Cai M., Kim S., Wang K., Farnham P.J., Coetzee G.A., Lu W. (2016). 4C-Seq Revealed Long-Range Interactions of a Functional Enhancer at the 8q24 Prostate Cancer Risk Locus. Sci. Rep..

[232] Luo Z., Rhie S.K., Lay F.D., Farnham P.J. (2017). A Prostate Cancer Risk Element Functions as a Repressive Loop That Regulates HOXA13. Cell Rep..

[233] Freedman M.L., Monteiro A.N.A., Gayther S.A., Coetzee G.A., Risch A., Plass C., Casey G., De Biasi M., Carlson C., Duggan D. (2011). Principles for the Post-GWAS Functional Characterization of Cancer Risk Loci. Nat. Genet..

[234] Zhang Z., Chng K.R., Lingadahalli S., Chen Z., Liu M.H., Do H.H., Cai S., Rinaldi N., Poh H.M., Li G. (2019). An AR-ERG Transcriptional Signature Defined by Long-Range Chromatin Interactomes in Prostate Cancer Cells. Genome Res..

[235] Zhang L., Yang S., Chen X., Stauffer S., Yu F., Lele S.M., Fu K., Datta K., Palermo N., Chen Y. (2015). The Hippo Pathway Effector YAP Regulates Motility, Invasion, and Castration-Resistant Growth of Prostate Cancer Cells. Mol. Cell. Biol..

[236] Coffey K. (2021). Targeting the Hippo Pathway in Prostate Cancer: What’s New?. Cancers.

[237] Yuan G., Flores N.M., Hausmann S., Lofgren S.M., Kharchenko V., Angulo-Ibanez M., Sengupta D., Lu X., Czaban I., Azhibek D. (2021). Elevated NSD3 Histone Methylation Activity Drives Squamous Cell Lung Cancer. Nature.

[238] Li W., Tian W., Yuan G., Deng P., Sengupta D., Cheng Z., Cao Y., Ren J., Qin Y., Zhou Y. (2021). Molecular Basis of Nucleosomal H3K36 Methylation by NSD Methyltransferases. Nature.

[239] Cabrera-Licona A., Pérez-Añorve I.X., Flores-Fortis M., Moral-Hernández O.D., González-de la Rosa C.H., Suárez-Sánchez R., Chávez-Saldaña M., Aréchaga-Ocampo E. (2021). Deciphering the Epigenetic Network in Cancer Radioresistance. Radiother. Oncol..

[240] Kvon E.Z., Waymack R., Gad M., Wunderlich Z. (2021). Enhancer Redundancy in Development and Disease. Nat. Rev. Genet..

[241] Ma X.-X., Cao Z.-G., Zhao S.-L. (2020). m6A Methyltransferase METTL3 Promotes the Progression of Prostate Cancer via m6A-Modified LEF1. Eur. Rev. Med. Pharmacol. Sci..

[242] Yuan Y., Du Y., Wang L., Liu X. (2020). The M6A Methyltransferase METTL3 Promotes the Development and Progression of Prostate Carcinoma via Mediating MYC Methylation. J. Cancer.

[243] Li E., Wei B., Wang X., Kang R. (2020). METTL3 Enhances Cell Adhesion through Stabilizing Integrin β1 mRNA via an m6A-HuR-Dependent Mechanism in Prostatic Carcinoma. Am. J. Cancer Res..

[244] Itkonen H.M., Minner S., Guldvik I.J., Sandmann M.J., Tsourlakis M.C., Berge V., Svindland A., Schlomm T., Mills I.G. (2013). O-GlcNAc Transferase Integrates Metabolic Pathways to Regulate the Stability of c-MYC in Human Prostate Cancer Cells. Cancer Res..

[245] Itkonen H.M., Poulose N., Steele R.E., Martin S.E.S., Levine Z.G., Duveau D.Y., Carelli R., Singh R., Urbanucci A., Loda M. (2020). Inhibition of O-GlcNAc Transferase Renders Prostate Cancer Cells Dependent on CDK9. Mol. Cancer Res..

[246] Itkonen H.M., Urbanucci A., Martin S.E., Khan A., Mathelier A., Thiede B., Walker S., Mills I.G. (2019). High OGT Activity Is Essential for MYC-Driven Proliferation of Prostate Cancer Cells. Theranostics.

[247] Chen S., Zhu G., Yang Y., Wang F., Xiao Y.-T., Zhang N., Bian X., Zhu Y., Yu Y., Liu F. (2021). Single-Cell Analysis Reveals Transcriptomic Remodellings in Distinct Cell Types That Contribute to Human Prostate Cancer Progression. Nat. Cell Biol..

[248] Zhang Z., Karthaus W.R., Lee Y.S., Gao V.R., Wu C., Russo J.W., Liu M., Mota J.M., Abida W., Linton E. (2020). Tumor Microenvironment-Derived NRG1 Promotes Antiandrogen Resistance in Prostate Cancer. Cancer Cell.

[249] Drilon A., Somwar R., Mangatt B.P., Edgren H., Desmeules P., Ruusulehto A., Smith R.S., Delasos L., Vojnic M., Plodkowski A.J. (2018). Response to ERBB3-Directed Targeted Therapy in NRG1-Rearranged Cancers. Cancer Discov..

[250] Sun Y., Campisi J., Higano C., Beer T.M., Porter P., Coleman I., True L., Nelson P.S. (2012). Treatment-Induced Damage to the Tumor Microenvironment Promotes Prostate Cancer Therapy Resistance through WNT16B. Nat. Med..

[251] Zadra G., Loda M. (2019). When Fat Goes Down, Prostate Cancer Is on the Ropes. Mol. Cell. Oncol..

[252] Butler L.M., Perone Y., Dehairs J., Lupien L.E., de Laat V., Talebi A., Loda M., Kinlaw W.B., Swinnen J.V. (2020). Lipids and Cancer: Emerging Roles in Pathogenesis, Diagnosis and Therapeutic Intervention. Adv. Drug Deliv. Rev..

[253] Kumaraswamy A., Welker Leng K.R., Westbrook T.C., Yates J.A., Zhao S.G., Evans C.P., Feng F.Y., Morgan T.M., Alumkal J.J. (2021). Recent Advances in Epigenetic Biomarkers and Epigenetic Targeting in Prostate Cancer. Eur. Urol..

[254] Doultsinos D., Mills I.G. (2021). Derivation and Application of Molecular Signatures to Prostate Cancer: Opportunities and Challenges. Cancers.

[255] Sjöström M., Zhao S., Small E.J., Ning Y., Maurice-Dror C., Foye A., Hua J.J.T., Li H., Beer T.M., Evans C.P. (2021). 5-Hydroxymethylcytosine as a Liquid Biopsy Biomarker in mCRPC. J. Clin. Oncol..

[256] Nakken S., Lilleby W., Switlyk M.D., Knudsen K.E., Lilleby O., Zhao S., Kaveh F., Ekstrøm P.O., Urbanucci A., Hovig E. (2021). The Quandary of DNA-Based Treatment Assessment in De Novo Metastatic Prostate Cancer in the Era of Precision Oncology. J. Pers. Med..

